# Supplementary material for: Nurture Early for Optimal Nutrition (NEON) programme: qualitative study of drivers of infant feeding and care practices in a British-Bangladeshi population
Source: BMJ Open. 2020 Jun 21;10(6):e035347. doi: 10.1136/bmjopen-2019-035347 (PMC7307527; doi:10.1136/bmjopen-2019-035347)
Supplement: Supplementary data [file bmjopen-2019-035347supp001.pdf]

Main theme	Sub-theme	Participants type	Example quotes
**Theme One: Modifiable Infant Feeding and Care Practices**	Early and late introduction of semi and solid foods	Mother	*"As a Bengali we tend to put our children onto solid food as quickly as possible".*
		Community member	*"[…] It's a cultural thing, that's what we lived on everyone, so everyone's parents say oh let's get them onto rice as quickly as possible. So that's the culture."*
		Grandfather	*"[…] most of the kids in Bangladesh they start solid food from 6 months old baby. They mash it and they give soft one. I believe in London as well they start at 6 months old and make it softer, make it mashed so children can easily eat. From 6 months they start a lot of movement and then it works to make them stronger."*
		Community member	*"What he is saying is, say for example right what you said is very, very true, what they try to do is some parents what they try to do, they give them for example breastfeeding then, 15 or 20 minutes later, the time has gone to go to sleep or something like that. Give them another bottle of milk.so obviously what*

		he's saying is overfeeding can be leading towards these kinds of problems in health issues because we just fed someone half an hour ago, why are we feeding the child again"
Overfeeding	Community member	*"The other thing is obviously a lot of the people say right in our community, which is true, people eat up to the brim of their neck and even sometimes you will see people eating just after 15 minutes before they go to bed which is not good because you have not digested your food yet, it is still in your chest."*
	Mother	*"The TV, yes he likes watching his teletubbies and stuff like that. If that is on then he sometimes does, rarely he eats, TV is his everything. If I am watching he comes up and take the remote from me. Smart like that, yes."*
	Mother	*"[…] you know some babies they don't like to eat, almost you have to make different circumstances to make them happy to eat, like some entertainment thing like rhymes or something. Or lots of toys to put in front of her high chair and then […]"*
	Health professional	*"Particularly we see lots of toddlers who are having excessive milk and not having very much solids because there's no real appetite for them […] No appetite, because they are always full of milk."*

	Feeding to fill the belly	Community member	*"I have heard comments like breast milk is too thin, it digests too quickly so that they need formula that will make their belly fuller as well, that is another indication that I have seen that they look for the belly to be sort of bulging a little bit."*
		Community member	*"There have been times, many, many times we've been telling parents how their baby's stomach is probably this small, like the size of their fist, that's how big your stomach is. So, if you are trying to feed them that much rice on a plate, that is more than they should be taking in. Parents tend to think oh no she didn't eat properly, she hasn't had 5 bites and they want to give more bites in her mouth or encourage to eat more. But really that is all the food she can take."*
		Mother	*"I never know, my husband always says that's enough, don't give no more. He likes you know Asians make this thing called Kheer, it is milk with rice and sugar and it is really sweet and it is really nice. He absolutely loves it. Any time of day you feed him that he'll eat it."*
		Mother	*"I just don't want him to have too much sweet, because obviously all the kids in my family had all teeth taken out. I don't want him to go through that, he's only*

		got five. I don't want him to lose all his beautiful teeth. I try and keep him away from sweets. I say that but I want to keep him away from sweet stuff”
	Community member	*“[…] they are all doing it you know and if we look at infants, I have seen them with bottles drinking it. I’ve seen the juice, I’ve seen coke in and like I said, to the schools I work in they are in the buggy and they have got a bottle of brown drink and we know that it is nothing but coke, so I almost feel sorry […]”*
	Mother	*“You put him in his highchair and give him a few spoons and that's it. No, turns his face. And I have to like literally beg him, please one more, one more, he won't eat. And he's quite a healthy child. You'd think he eats quite a bit, he doesn't. He drinks 8ozs of milk and for him that's enough, he doesn't want no more.”*
	Mother	*“[…] especially in the Bangladeshi community they have a Vitamin D deficiency so mum milk has everything but if the mum doesn’t have enough vitamin D, the baby is not going to get enough vitamin D, so doctors are saying that formula milk has vitamin D, so mixed feeding is good. I talk to the doctor and my health visitor, they all listen to me. The formula milk has vitamin D specially made for the deficiency and so yes, I need to give him formula milk.”*

Prolonged parent-led feeding practices	Community member	*"I think because my Mum's always hand feed us, even when we were quite old she would hand feed us, […] I'm sure that Mum's here have hand fed their […] my son is five years old and I will hand feed him because rice and curry is mostly hand fed. Also, it's the love as well, it's the love aspect. It's like you're showing them you love your child, so then you feed them more. More than the love aspect is the easiness […] instead of them making a mess you just feed them […]"*
	Mother	*"Culturally we've always eaten rice by hand, okay. That's something I would never let go of. In all honesty, rice and curry is so tasty with hands, we don't enjoy it with fork and knife."*
	Mother	"I saw most of the mothers running behind children with a spoon"
	Father	*"My boy is used to the bottle from his father, maybe at just one or two months he did breastfeeding, after that he didn't breast feed now he is sleeping my wife tried the breastfeeding at night time. Sometimes he drinks but sometimes not, but he used to have the bottle and it's easier for us as well to give him the bottle."*

		Health professional	*"A third of our children are on the SEN [special educational needs] Register which is speech and language. They are still probably having bottles, they are probably still on dummies and they probably aren't eating solid foods properly and they are being (hand) fed as well. That's the other thing in our community as well that children up until the age of about 7 or 8, even older, are physically fed rice, you know, hand fed. When children come into nursery… some of them don't even have a clue about how to use a knife and fork because they have been fed. When the food is fed… rice is mushed with their hand… it doesn't give them that whole experience of the chewing."*
		Health professional	*"It is almost a cultural thing in our society now that we let children have bottles for longer, one it is the oral health thing, you know if they are having sugary drinks via a bottle which is also wrong for their teeth, dental decay."*
	Influences from society and culture	Mother	*"If we try to talk to her about [overfeeding her child] she gets offended. In Bengali culture, we have what we call the 'evil eye' […] so, she thinks we are giving her children the 'evil eye' because they eat well, they eat a lot. We still talk to her about it [overfeeding] though, we don't stop."*

	Key Informant	*"Another thing, which is quite true actually, here now for example right, it's a cultural thing. Back home right for example, if you got a pot belly for example, if you got, they think you are healthy."*
	Pregnant woman	*"The baby who has good health with no disease, is chubby. With good health they look nice."*
Physical and local environment	Community member	*"If you want to be healthy you've got to start from the beginning. Here what do you do? People are living in a box, just in the house or flat, got very limited space for a young child, 9 months or 1-year old child, it's time for them to crawl around. Is there any space to do that? No. Their bone structure, everything, is not getting stronger because they are not exercising, they are not moving around that much. What they do, say put them in a pushchair, in front of the TV and that's it."*
	Grandfather	*"[…] my grandchild is nearly 6 years old and we did try a little bit of chips to taste it."*
	Community member	*"[…] I think anyway if you go to the supermarket and you see something that says four months then you should be doing that because the packaging, if the baby food companies are telling you that you should be feeding your baby at*

***Theme two:* Factors influencing infant feeding and care practices in the British-Bangladeshi population**		*four months why wouldn't you, why would you wait until they were six months, because even though it is not that much of a difference but when you have had a baby they are growing so quickly you think that two months is a big deal and that you should be giving that extra food, but definitely I do not think many families that I have seen will wait until six months."*
	Key Informant	*"[…] Especially around holiday time everyone goes to everyone's houses and things like that. A lot of the dishes would be made, obviously when they are doing these kinds of things you are not looking at what you are cooking, what kind of ingredient is going in, what level of salt or sugar has gone in. That is when children do eat quite a lot more than what they are supposed to eat when you have got guests coming into your house they are bringing like sweets, crisps and things like that for your children."*
	Mother	*"I am always confused, I am always ringing up the doctors and asking them questions all the time, especially about the milk thing. They said it depends on your child, every child is different. I was a bit like he's still little, doesn't he need the nutrients in the powder to grow?"*

Information and awareness	Mother	*"By starving, I actually used that term, starving may mean they think the child has not eaten [...] like for example if they have not eaten in two or three hours [...] I will give you a perfect example, I went to the wedding over the weekend and my mother in law was on my case to feed my son... I just said when he is hungry he can communicate with me, he will come up to me and say mum I want to eat. I tried three times, after three times I am not going to go round walking after him. My mother in law you know how she is she is so protective of her grandchildren[...] she took him and she fed him, so can you see the difference?"*
	Mother	*"I remember before I got married before I had my kids they used to do what my mum did which my mum did twenty-three years ago, twenty four years ago, at that time it was just egg custard and stuff so they did not know much about vegetables, they did now know much about a lot of stuff. Even when we were growing up probably my mum gave like at six months they started introducing what they ate but before that they gave more like egg custards"*

	Grandmother	*"[If] they are fussy eater, it is just because they were fed on only milk because of my daughter's in laws, they are afraid that house will be a mess. They do not know the foods, you have to try foods to know."*
	Mother	*"Yes, I am worried about him because he's not eating. I try and give him, if he eats anything like the other day I gave him a choc chip roll when I shouldn't have, like it's not good for him to have and he ate it and I was so happy. He never says no to chocolate though, that's a bad thing. Milky bar, his grandparents, always milky bars, he loves it."*
	Mother	*"I notice in our community we have a real thing about feeding them and making sure the fact they are having a full ... do you see what I mean but I just tend to find that be a bit relaxed, see if they take to it, if they eat or whatever. If they don't eat, come back to it, come back to the same meal, come back to that thing. It might be you try something else, they might like it but don't become stressed by it."*
