# Supplementary material for: Nurture Early for Optimal Nutrition (NEON) programme: qualitative study of drivers of infant feeding and care practices in a British-Bangladeshi population
Source: BMJ Open. 2020 Jun 21;10(6):e035347. doi: 10.1136/bmjopen-2019-035347 (PMC7307527; doi:10.1136/bmjopen-2019-035347)
Supplement: Author's manuscript [file bmjopen-2019-035347.draft_revisions.pdf]

BMJ Open is committed to open peer review. As part of this commitment we make the peer review history of every article we publish publicly available.

When an article is published we post the peer reviewers' comments and the authors' responses online. We also post the versions of the paper that were used during peer review. These are the versions that the peer review comments apply to.

The versions of the paper that follow are the versions that were submitted during the peer review process. They are not the versions of record or the final published versions. They should not be cited or distributed as the published version of this manuscript.

BMJ Open is an open access journal and the full, final, typeset and author-corrected version of record of the manuscript is available on our site with no access controls, subscription charges or pay-per-view fees (http://bmjopen.bmj.com).

If you have any questions on BMJ Open's open peer review process please email info.bmjopen@bmj.com

BMJ Open

Nurture Early for Optimal Nutrition (NEON) Programme: Qualitative study of drivers of infant feeding practices in a British-Bangladeshi population

Journal:	*BMJ Open*
Manuscript ID	bmjopen-2019-035347
Article Type:	Original research
Date Submitted by the Author:	29-Oct-2019

Complete List of Authors:	Benton, Lorna; University College London Institute of Child Health, Population, Policy and Practice<br>Lloyd, Oliver ; University College London Institute of Child Health, Population, Policy and Practice<br>Heys, Michelle ; University College London Institute of Child Health, Population, Policy and Practice<br>Manikam, Logan; University College London Institute of Epidemiology and Health Care, Department of Epidemiology and Public Health<br>Rosenthal, Diana; University College London Institute of Child Health, Population, Policy and Practice<br>Lakhanpaul, Monica; University College London Institute of Child Health, Population, Policy and Practice

Keywords:	Community child health < PAEDIATRICS, NUTRITION & DIETETICS, PUBLIC HEALTH

SCHOLARONE™ Manuscripts

I, the Submitting Author has the right to grant and does grant on behalf of all authors of the Work (as defined in the below author licence), an exclusive licence and/or a non-exclusive licence for contributions from authors who are: i) UK Crown employees; ii) where BMJ has agreed a CC-BY licence shall apply, and/or iii) in accordance with the terms applicable for US Federal Government officers or employees acting as part of their official duties; on a worldwide, perpetual, irrevocable, royalty-free basis to BMJ Publishing Group Ltd ("BMJ") its licensees and where the relevant Journal is co-owned by BMJ to the co-owners of the Journal, to publish the Work in this journal and any other BMJ products and to exploit all rights, as set out in our licence.

The Submitting Author accepts and understands that any supply made under these terms is made by BMJ to the Submitting Author unless you are acting as an employee on behalf of your employer or a postgraduate student of an affiliated institution which is paying any applicable article publishing charge ("APC") for Open Access articles. Where the Submitting Author wishes to make the Work available on an Open Access basis (and intends to pay the relevant APC), the terms of reuse of such Open Access shall be governed by a Creative Commons licence – details of these licences and which Creative Commons licence will apply to this Work are set out in our licence referred to above.

Other than as permitted in any relevant BMJ Author's Self Archiving Policies, I confirm this Work has not been accepted for publication elsewhere, is not being considered for publication elsewhere and does not duplicate material already published. I confirm all authors consent to publication of this Work and authorise the granting of this licence.

For peer review only

Title

Nurture Early for Optimal Nutrition (NEON) Programme: Qualitative study of drivers of infant feeding practices in a British-Bangladeshi population

Authors and affiliation

1. Lorna Benton

   Population, Policy and Practice, University College London Institute of Child Health, London, UK

2. Oliver Lloyd

   Population, Policy and Practice, University College London Institute of Child Health, London, UK

3. Michelle Heys

   Population, Policy and Practice, University College London Institute of Child Health, London, UK

4. Logan Manikam,

   Department of Epidemiology and Public Health, University College London Institute of Epidemiology and Health Care, London, UK

5. Diana Margot Rosenthal

   Population, Policy and Practice, University College London Institute of Child Health, London, UK

6. Monica Lakhanpaul

   Population, Policy and Practice, University College London Institute of Child Health, London, UK

Corresponding author:

Monica Lakhanpaul

For peer review only - http://bmjopen.bmj.com/site/about/guidelines.xhtml

Professor of Integrated Community Child Health, UCL Great Ormond Street Institute of Child Health, London WC1N 1EH.

E-mail: m.lakhanpaul@ucl.ac.uk

Telephone number: +442079052259

Author Contributions: All authors worked equally on it.

Word Count: 7,707 (ex title page, abstract, references, figures and tables)

For peer review only

Abstract

Objectives: To explore optimal nutritional care practices and their drivers within the British Bangladeshi population of East London as an exemplar to inform a tailored, co-adapted intervention design.

Design: Qualitative community based participatory research

Setting: Community and children's centres and NHS settings within Tower Hamlets

Participants: A total of 145 participants completed the study, including; British Bangladeshi mothers, fathers, grandmothers and grandfathers of infants and young children aged 6-23months; key informants and lay community members from the British Bangladeshi population of Tower Hamlets and health professionals working in Tower Hamlets.

Results: Participants discussed several infant feeding practices that may restrict dietary diversity, override infant satiety and encourage dependence on sugary or sweetened foods and impact on broader care practices such as sleeping and physical activity. Specifically, four modifiable infant feeding and care practices were highlighted: untimely introduction of semi- and solid foods, overfeeding, prolonged parent-led feeding and feeding to 'fill the belly'. These practices were driven by factors at all socio-ecological levels, categorised as; society, culture and religion, physical and local environment and information and awareness.

Conclusions: Parenting interventions need to be culturally sensitive to populations of diverse ethnicity and recognise the social and cultural norms that inform complementary feeding practices. Further, the UK infant feeding environment requires better regulation of marketing of foods for infant and young children. More evidence is needed to inform effective early years interventions.

For peer review only - http://bmjopen.bmj.com/site/about/guidelines.xhtml

Keywords: British Bangladeshi; Nutrition; Children; Infant; Feeding Practice; Early Interventions ; Complementary Feeding

Article summary

Strengths and limitations of this study

- Existing literature does not adequately capture the cultural and social determinants of infant feeding and care.
- A strength of this study is the development of culturally sensitive interview tools and recruitment strategies.
- This strong partnership with members of the British-Bangladeshi population of Tower Hamlets enabled further reach and diversity of representation in this study.
- A limitation of this qualitative study is that a quantitative measure of dietary intake was out of scope.
- Another limitation is that all data are self-reported and have the potential for respondent bias.

For peer review only

Nurture Early for Optimal Nutrition (NEON) Programme: Qualitative study of drivers of infant feeding practices in a British-Bangladeshi population

INTRODUCTION

The first 1,000 days of life presents a critical window to prevent the dual burden of under- and over-nutrition. In the UK, an estimated 5% of the population is undernourished, of which 70% goes unreported [1]. Meanwhile, obesity prevalence continues to rise in accordance with projections that over half of UK adults could be obese by 2050 [2]. Currently, approximately one third of children leave primary school as obese or overweight in the UK [3] with substantial implications for: underachievement in school, lower self-esteem and risk of developing cardiovascular disease or type 2 diabetes into adulthood [4].

Health inequities contribute to the risk of nutritionally related diseases across the life course, such as coronary heart disease and allergy [5-11]. Prevalence of childhood obesity is twice as high amongst those living in the most deprived, compared to the least deprived areas of the UK [12] and a similar trend is observed elsewhere in Europe [13]. Some of the highest burdens of inequity and obesity are measured amongst ethnic minority populations but a tendency to aggregate data from South Asian populations limits a full understanding of trends that result from different socio-cultural norms and practices. Studies that more specifically examine nutritional outcomes in the British-Bangladeshi population have reported an increased risk of vitamin D deficiency [14] and iron deficiency [15] in children, the highest proportion of obesity (31%) of any ethnic group amongst 6-year-old boys [16] and poorer oral health in pre-school children [17].

Interventions delivered in the early years, specifically within the critical first 1001 days, to optimise nutrition could provide a holistic and potentially cost-effective approach to the prevention of nutritionally related diseases [18]. A wide literature base exists on barriers to exclusive breastfeeding, yet

For peer review only

comparably little attention has been offered to complementary feeding practices. The 6-23-month age period is known as the 'weaning' or 'complementary feeding' age, when solid and semi-solid foods are nutritionally required to support breastfeeding. In 2018 the Scientific Advisory Committee on Nutrition (SACN) published the much-anticipated 'Feeding in the First Year of Life' report. Although many recommendations remained unchanged, it recognised the importance of feeding practices and caregiver behaviours, such as familiarising infants to a range of flavours and textures [19].

One forthcoming challenge is how to translate SACN guidelines into effective infant feeding programmes. Evidence demonstrates that culturally sensitive infant and young child feeding (IYCF) programmes need to be tailored to the target population and inclusive of the extended family and friends that typically offer more regular and often traditional support [20]. Although the NHS has long recognised the value of community-centred approaches, few studies explore cultural and social drivers of complementary feeding practices to inform models that utilise local resources and assets [21,22]. This is an opportunity to learn from low-resource approaches that have proven to be effective globally [23-25] and build local partnerships to develop tailored community-based approaches. The results in this paper represent the formative phase of a wider programme of work, designed to develop generalizable evidence that can be ultimately applied to optimise infant feeding practices in the British Bangladeshi population, following an adapted Participatory Learning and Action approach.

For peer review only

METHODOLOGY

Study design

NEON (Nurture Early for Optimal Nutrition) is a National Institute for Health Research funded study that aims to explore common complementary feeding practices and their social and cultural influencers within the British-Bangladeshi population of Tower Hamlets. NEON follows a sequential design for evidence generation, beginning with a systematic review series of the literature on infant feeding practices in Bangladeshi and other South Asian families living in: India, Pakistan, Bangladesh and high income setting [26-29]. This paper presents the results of a qualitative formative study phase, with participants from 'community', 'key informant', 'health professional' and 'family' groups, as informed by the multiple levels of the socio-ecological framework. Evidence from the literature and qualitative study informed an adapted Participatory Learning and Action (PLA) group approach to optimise infant feeding and care practices amongst Bangladeshi infants aged 6 to 23 months and will be described elsewhere.

A Community Based Participatory Research Partnership

Informed by a Community-Based and Participatory approach [30,31], Community Facilitators (CF) were active partners in study design, data collection, analysis and interpretation, towards intervention development. Community facilitators worked as partners in the study team and as a bridge to the community, helping to facilitate engagement, crossing language barriers and informing accessible and appropriate topic guides. Patient Public Involvement (PPI) representatives were invited to attend quarterly meetings and provide cultural oversight and study accountability in more of a consultation capacity. CF and PPI roles were advertised through local community organisations and parent networks. CF/PPI recruitment criteria were a) adult over 18 years of age, b) parent, c) self-identified as being of Bangladeshi heritage and d) live in local area. Tower Hamlets at time of study. Two CFs (one male, one female) and two PPI (both female) were selected via interview according to their awareness and motivation towards the study and topic. CFs received an orientation day, a

For peer review only - http://bmjopen.bmj.com/site/about/guidelines.xhtml

refresher day and two days of full training on interview and facilitation skills, data confidentiality and informed consent. CF's and PPI received high street vouchers for their involvement and travel costs. The value varied according to length of the activity and guidance from community-based research partnership experts and Involve. The lead researcher (LB) holds a PhD, is female and was employed at University College London for the duration of this study.

Study Population

The study is located in the London Borough of Tower Hamlets (TH), an area with the largest British Bangladeshi population in the UK and one of the most socio-economically deprived of any London borough, scoring the highest rates of child poverty and unemployment [32]. Sylheti is the most common language spoken by British Bangladeshi residents of TH but is not a written language, meaning health promotion materials are usually written in English or Bengali. This body of work will further generate transferable lessons to inform similar studies for adapted or tailored programmes with other vulnerable populations.

Participant recruitment

Multiple recruitment strategies were used to purposively sample the British Bangladeshi population of TH. CFs shared study details with local schools, community and children's centres, NHS clinics within different districts ranging from Whitechapel to the Isle of Dogs. To ensure access to families that might not otherwise access health services, CFs also actively recruited from public spaces and through informal networks and through word-of-mouth. Posters were developed in English and Bengali and placed in public spaces, however, CFs were encouraged to recruit via word of mouth to overcome language and literacy barriers. Key Informants were identified during community focus-group discussions (FGDs) and through snowball sampling. Health professionals were identified and contacted by the researchers. Potential participants were approached by CFs in person or via telephone and provided with information and expression of interest documents. CF's arranged a venue, time and date convenient to participants, utilising community centres, children centres and

For peer review only - http://bmjopen.bmj.com/site/about/guidelines.xhtml

other public spaces in Tower Hamlets. The researcher organised all financial costs related to room bookings, transport and snacks.

Sampling and inclusion

Study participants were eligible if they self-identified as British Bangladeshi and were resident to TH, with the exception of health professionals who were only required to practice in TH. Inclusion criteria further differed by participant type. Community members were not required to have children in the study age range (6-23months). Community key informants were included on the criteria that they held a significant role in the community. Health professionals were eligible if they had experience of nutritionally related diseases amongst infants and young children in TH; professions represented in this study include health visiting, midwifery, speech and language therapy, dentistry and GP. Finally, participants of the family phase were eligible if a) pregnant or b) caring for a child aged 6-23months in the capacity of: mother, father, caregiver or grandparent. Pregnant women and mothers were stratified according to time spent living in the UK (< or > 3years).

Focus groups

FDGs were first conducted with community members to explore social norms towards infant feeding and identify stakeholders influencing infant feeding practices in the target population. FGDs were later conducted with fathers and grandparents to gain a consensus of understanding and inform findings from semi-structured interviews (SSI) conducted with mothers and pregnant women. As advised by CF/PPI, all FGDs were separated by gender and status, i.e. 'fathers', 'grandmothers' and 'grandfathers' to ensure a comfortable environment for discussion. FGDs were co-facilitated by the researcher (LB) and a CF. The gender of the CF was selected to match that of the FGD participants. One person acted as facilitator whilst the other took observational notes and this varied according to study phase and group. At first the researcher facilitated the groups but as the study progressed, CFs were capable to run the FGD and the researcher observed. The researcher led the informed consent in English and CF's translated into Bengali or Sylheti. Focus groups were conducted in community centres around

For peer review only - http://bmjopen.bmj.com/site/about/guidelines.xhtml

Tower Hamlets and lasted approximately 90-120mins. Audio recordings were taken for transcription and translation and all recorded interviews were then destroyed.

Semi-structured Interviews (SSI)

SSI were conducted with key informants, mothers, pregnant women and healthcare professionals because this approach allows a more in-depth exploration of infant feeding practices and influencers amongst target beneficiaries of this study. We sought to explore in more depth cultural and social norms towards infant feeding. These norms may change after migration to a new country [33] and thus we stratified recruitment of mothers and pregnant women by migration status: based in the UK <3 years or >3years. The CF typically led key informant, mother and pregnant women SSIs in English, Bengali, Sylheti or a mixture of languages in community spaces or in the person's own home. Health professional interviews were conducted in English and in an NHS space or via phone, by the researcher (LB). SSIs were conducted individually except for one mother interview, where her mother and sister also attended. SSIs lasted approximately 45-90minutes and were audio-recorded.

Topic guides

Topic guides followed a consistent structure but were tailored to the participant to encourage a progressive depth of information gained. The literature [26-29] informed community and key informant and health professional interview tool guides. 'Family' topic guides were modified based on findings from these prior phases and tailored to family member (e.g. pregnant woman or mother). Participants were asked their thoughts on what foods they consider as healthy, and what a healthy child looks like and why, except for health professionals, who discussed influencing factors and health consequences encountered in their profession. Prompts were prepared and used for social, cultural and environmental influences on infant feeding. Guides were developed in English and translated into Bengali. Translations were checked and edited CFs to ensure reliability and consistency. CFs were prepared to speak in Sylheti, Bengali or English, as per the needs of the participants.

For peer review only - http://bmjopen.bmj.com/site/about/guidelines.xhtml

Film

Stories and anecdotes collected during the early phases of data collection were used to produce a short film, introducing the topic of the study and including stories to parents and caregivers that might be otherwise difficult to share to e.g. the narrative from one mother of filling a bottle of milk with crushed biscuits to 'top up' her infant feeding. The film was used to help with ice breaking during individual interviews with parents and pregnant women. The use of film has been recognised as a useful tool to support participants in feeling less threatened whilst yet familiarising them with the issue to be discussed.

Ethical considerations

During FGDs and interviews, the researcher led the informed consent in English and community facilitators translated into Bengali or Sylheti. The project was approved for research ethics from the University College London Research Ethics Committee (Ethics project ID number: 10271001)

Qualitative Data Analysis

Transcripts were checked by the researcher for fidelity to the topic guide and detail of prompts as data collection progressed. The steps of a framework analysis process were followed, as outlined by Braun and Clarke [34] and included: transcription, familiarisation with the interview, coding, developing a working analytical framework, applying the analytical framework, charting data into the framework matrix and interpreting the data. Transcriptions were completed by CF and outsourced to a professional company, parallel to data collection. CF and LB discussed and agreed when data saturation had been achieved. A combined inductive and deductive approach was taken to allow for socially located themes in the assumption that unspecified traditional and cultural beliefs are likely to influence feeding practices in this population. Two researchers independently familiarised themselves with the data and discussed preliminary themes and codes, to decide on the final themes and subthemes. Researchers applied open codes to the transcripts using NVivo 11 [35] and sought CF input into thematic analysis, to revise themes where applicable.

For peer review only - http://bmjopen.bmj.com/site/about/guidelines.xhtml

RESULTS

A total of 145 participants were recruited to 12 focus groups and 45 semi-structured interviews (Table 1).

Table 1: Characteristics of study participants

Participant	FGD / SSI	Sex	N (Total)	Language
Community	FGD (n=6)	Female	7	Sylheti
		Male	8	English with some Sylheti
		Female	5	English with some Sylheti
		Female	10	Sylheti
		Male	7	English with some Sylheti
		Female	10	English with some Sylheti
Key Informants	SSI	Mixed	6	Mixed
Health professionals	SSI	Mixed	9	English
Mothers	SSI	Female	21	Mixed
Pregnant women	SSI	Female	9	Mixed
Fathers	FGD (n=2)	Male	6	Mixed
		Male	4	Mixed
Grandmothers	FGD (n=2)	Female	14	Mixed
		Female	14	Mixed
Grandfathers	FGD (n=2)	Male	6	Mixed
		Male	5	Mixed

FDG: Focus discussion group

SSI: semi-structured interview

For peer review only - http://bmjopen.bmj.com/site/about/guidelines.xhtml

Two overarching themes were identified during interviews and group discussions: (1) modifiable infant feeding practices that participants suggested could be targeted in order to optimise infant feeding and (2) socio-ecological factors believed by participants to influence these modifiable feeding practices.

For peer review only

For peer review only - http://bmjopen.bmj.com/site/about/guidelines.xhtml

Theme One: Modifiable Infant Feeding and Care Practices

Figure 1 depicts the four subthemes identified, namely: ‘early and late introduction of semi and solid foods’; ‘overfeeding’; ‘feeding to fill the belly’ and ‘prolonged parent-led feeding practices’. Within some of these subthemes there were further connecting sub-themes as depicted in Figure 1. For example, overfeeding included four connecting sub-themes: forced feeding, unregulated portioning, top-up feeding and distraction feeding.

Figure 1: Connecting sub-themes related to Theme One: Modifiable Infant Feeding and Care Practices

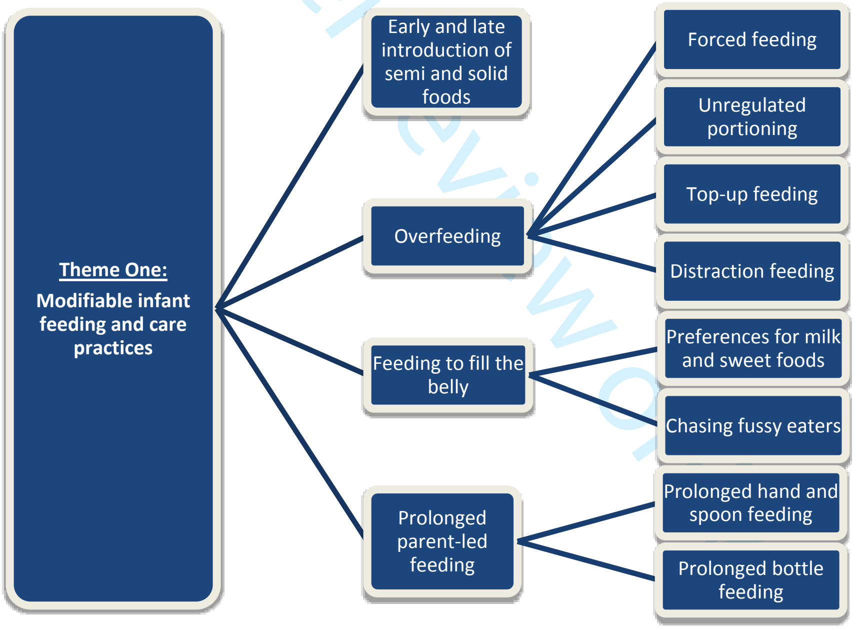

Early and late introduction of semi and solid foods

Parents were highly aware of NHS recommendations to introduce semi-solid and solid foods at six months. We identified differing attitudes towards this recommendation, favouring early (3-5 months), timely (6months) and late (7-8 months) in different households and a sentiment that specific advice regarding

For peer review only - http://bmjopen.bmj.com/site/about/guidelines.xhtml

timing is '*always changing'*. Early introduction was favoured amongst many participants to improve the nutrition of non-breastfed babies or help infants to grow and develop faster. Food advertising was given as a reason for the early introduction of solids from four months of age in a few households. Conversely, only few parents introduced solid and semi-solid foods later than 6 months and did so because they perceived milk as nutritionally sufficient. Late weaning was not as typical as early weaning.

"*As a Bengali we tend to put our children onto solid food as quickly as possible*".

-Mother

Reasons cited by caregivers to introduce solid foods (irrespective of timing of introduction) included: the personality of child, showing interest in food, health professional advice, when infant starts putting things in their mouth and when the infant is sitting up or crying for food.

Overfeeding

Awareness on how to regulate feeding portions was mixed, with some parents stating that infants are able to self-regulate and others stating they '*don't have the understanding of fullness*'. Four approaches to feeding are described below under the sub-theme of overfeeding.

Forced feeding

Key informants and health professionals defined "force feeding" as consistently encouraging a child to eat when not hungry. Parents did not report force-feeding themselves, but some said they had seen examples in their family or community. For example, holding a child in arms and feeding via parent led practices, when the child is not thought to be hungry. As a nuance to the theme, several mothers and fathers reported parents need to '*work hard and not force the child*' because it can be an unpleasant or stressful experience and risks making children afraid of food. Those parents said they stopped feeding when the infant/child pushes the spoon away, refuses food, says 'no' or closes their mouth.

For peer review only - http://bmjopen.bmj.com/site/about/guidelines.xhtml

Un-regulated portioning

Family and community participants agreed that portion-size is not customarily measured and often based on intuition. Health professionals reported that portion size can reflect adult portioning and that parental awareness, and a high frequency of feeds are barriers to age appropriate portioning. Parents felt that children were not eating enough volume of foods when they take 'a half portion' or '2-3 spoons' per feed. One health professional addressed this concern in their own clinical practice (unrelated to this study) by filming in several households and measuring portion size and comparing perceived portion size with measured portions size. They found these differed with perceived portion size frequently underestimating actual portion size (personal communication). Exceptions were found, however, amongst some parents, usually those that had received support from health services or sought information online.

Top-up feeding

Regular 'top-up' feeding is defined as the practice of offering frequent and additional meals to an infant's feeding routine. Meal timings varied between households with a tendency to eat late into the evening (8pm or later), providing time for extra feeds before infant/child bedtime. Some parents in this study said they gave their children milk and sometimes solid (jar) food immediately before the children go to bed. . Occasionally, caregivers reported adding sugar or biscuits to milk bottles to encourage calorie intake between solid feeds.

"The other thing is obviously a lot of the people say right in our community, which is true, people eat up to the brim of their neck and even sometimes you will see people eating just after 15 minutes before they go to bed which is not good because you have not digested your food yet, it is still in your chest."

- Community member

Distraction feeding

Distraction feeding describes the caregiver's practice of distracting the infant/child with videos on the phone or TV whilst feeding directly, e.g. via hand or spoon. Parents reported mixed attitudes towards the importance of play

For peer review only - http://bmjopen.bmj.com/site/about/guidelines.xhtml

during meal times: some felt they benefitted by avoiding messy eating and from the convenience of using distraction techniques, whilst others viewed this as a missed opportunity for interaction between caregiver and child. The TV was perceived as a helpful tool for grandparents with limited mobility to spend time with a child and supported mothers to undertake other household chores. In contrasting reports, participants also described engaging meal time activities including singing and storytelling, allowing or encouraging the infant to make a mess at meal times.

"She did not have the freedom to let her child play with food or eat with themselves because of her in laws even at her own house. And now they are fussy eaters, because they were restricted in how they can eat"

- Community member

Feeding to fill the belly

Community and family members described how caregivers often feel a need to *'fill the belly'*, to ensure good growth and development. Some health professionals elaborated on this theme from their experience of parents that place an emphasis on feeding 'enough'. At most extreme, this can be taken as a literal demonstration in which community members said that caregivers might look for a physical protrusion of the belly. Awareness of fussy eaters appeared to motivate some parents to practice a range of approaches during mealtimes.

Preferences for milk and sweet foods

A desire to fill the belly influenced the types of food and drink that parents preferred to give their infants, introducing formula milk, high energy (prioritising rice-based foods) and sugary snacks (e.g. yoghurt, banana, crisps or other snacks).

"I have heard comments like breast milk is too thin, it digests too quickly so that they need formula that will make their belly fuller as well, that is another

For peer review only - http://bmjopen.bmj.com/site/about/guidelines.xhtml

indication that I have seen that they look for the belly to be sort of bulging a little bit."

- Community member

Community members, informants and some parents recognised sugar as a customary part of the British-Bangladeshi diet. Informants described a tradition to provide a date as the first food. Families described how sweets and treats are customarily shared by household visitors and health professionals addressed the consequence that treats are frequently visible or on display in the household. Mothers reported treats, including custard tarts and biscuits or fizzy drinks, are commonly shared with young children.

"I just don't want him to have too much sweet, because obviously all the kids in my family had all teeth taken out. I don't want him to go through that, he's only got five. I don't want him to lose all his beautiful teeth. I try and keep him away from sweets. I say that but I want to keep him away from sweet stuff"

- Mother

Negative attitudes to fizzy drinks prevailed amongst study participants and very few people reported giving them to very young children. Mothers reported regularly preparing fruit juices, either as fresh, using a blender, or purchased, for example mango juice from concentrate. Caregivers considered homemade smoothies and fruit juices as a healthy breakfast, lunch or snack but health professionals connected these preparations to a high daily sugar intake. Health workers described a tendency to wean onto the bottle using fruit juices and fruit squashes. More informed participants were aware of the risk to oral health posed by sugary diets, but many other parents felt unable to break the dependence on sugar.

For peer review only - http://bmjopen.bmj.com/site/about/guidelines.xhtml

Chasing fussy eaters

The phenomena of fussy eaters were described by participants as: children running away from or denying food, avoiding vegetables, avoiding solids, sticking to one kind of food or only taking three spoons against an expectation of four or five. Commonly described scenarios were of parents following children around, trying to feed them with their hands. Self-proclaimed parents of fussy eaters described their anxiety and concern for the dietary diversity of their children.

"You put him in his highchair and give him a few spoons and that's it. No, turns his face. And I have to like literally beg him, please one more, one more, he won't eat. And he's quite a healthy child. You'd think he eats quite a bit, he doesn't. He drinks 8ozs of milk and for him that's enough, he doesn't want no more."

- Mother

Parents shared a range of concerns around the potential future health of their child and some spoke to the risk of Vitamin D and iron deficiencies when asked if they provide their child with any supplementation. A concern around the risk of Vitamin D deficiency stimulated one mother to switch to formula milk. Respondents from the community, health professional and family groups pointed to a cycle of fussy eating and the consumption of milk rather than solid foods. Health professionals observed that meal times can last around an hour, or could be replaced by sugary snacks and treats, perpetuating a cycle of milk and sugar but no meal. They recommended early exposure to a variety of textures and repeated attempts to introduce new foods (for example, they described routinely advising families that it can take up to 14 attempts to accept a new food item) and advised against distraction feeding as a risk towards over-feeding.

For peer review only - http://bmjopen.bmj.com/site/about/guidelines.xhtml

Prolonged parent-led feeding practices

Infant feeding practices were predominantly parent-led (hand feeding, spoon-feeding, bottle-feeding, syringe) with few parents practicing infant-led (i.e. with finger foods) feeding.

Prolonged hand and spoon-feeding

Hand feeding was practiced by parents as a symbol of love from parent to child and because they felt it could improve the taste of food whilst also allowing parents to remove small bones and to avoid messy feeding. Health professionals and some parents raised concerns for extensive hand and spoon-feeding over a prolonged period because it is a missed opportunity to encourage self-feeding practices and cognitive-motor skill development. The immediate social impact was evident in a commonly described scenario, where some British Bangladeshi children are unable to use knives and forks when they reach primary school.

"Culturally we've always eaten rice by hand, okay. That's something I would never let go of. In all honesty, rice and curry is so tasty with hands, we don't enjoy it with fork and knife."

- Mother

Spoon-feeding was preferred for semi-solids, such as porridge or kichadi (a rice and lentil preparation) or during teething, whilst the syringe was less commonly used and mostly for water during digestion problems. Those using beakers were also following advice to introduce at 6 months and often use it for water or juices such as Ribena. Infants were fed in a range of positions, often depending on the method of feeding and location. Inside the home infants may sit in a high chair at the table or separately, on the lap, floor, sofa or caregiver's arms. A common scenario was described of '*chasing the baby around the sofa*', although this was seen as less common.

For peer review only - http://bmjopen.bmj.com/site/about/guidelines.xhtml

" I saw most of the mothers running behind children with a spoon"

- Mother

Prolonged bottle feeding

Bottle-feeding is the norm amongst British-Bangladesh families in this study and, as participants indicated, in British families more broadly. Parents reported that the bottle is introduced as early as a few weeks or months in households that followed a mixed (breast and bottle) feeding approach. Occasionally parents were motivated by a belief that formula milk is scientifically prepared and thus more beneficial for child. Many mothers introduced the bottle after birth to facilitate feeding outside of the house, during social situations or when visitors come to their home. Typically, the bottle was viewed as a stepping-stone towards introduction of solid foods. Friends and family often encouraged bottle-feeding to ensure the 'belly is full', as it allows the possibility of more frequent feeds, such as an extra feed before bed to help the infant/child sleep and allows family members to help free up the mother's time. There were various accounts of how community members had seen the bottle being used inappropriately, for example, the contents being modified to include sugary additives, including biscuits, or to give juice such as mango to infants less than 6 months of age. One member recalled a friend had left a bottle of juice in the mouth of an infant overnight, something that she was concerned about for the impact that could have on tooth decay. Another described 'modified bottle' practice included cutting the teat of the bottle to feed semi-solid foods.

One health professional recommended a complete transition away from the bottle by 1 year of age, but a form of prolonged bottle-feeding was described amongst children up to the age two or three years old in many of the households. A speech and language professional identified prolonged sucking on a bottle teat as a risk factor for impeded devolvement in the roof of the mouth of some British-Bangladeshi infants in their care. Health professionals also expressed concern that prolonged dependence on the bottle may suppress the appetite and

For peer review only - http://bmjopen.bmj.com/site/about/guidelines.xhtml

that it is linked with an additional issue of dietary diversity of children consuming excessive milk.

"A third of our children are on the SEN [special educational needs] *Register which is speech and language. They are still probably having bottles, they are probably still on dummies and they probably aren't eating solid foods properly and they are being (hand) fed as well. That's the other thing in our community as well that children up until the age of about 7 or 8, even older, are physically fed rice, you know, hand fed. When children come into nursery… some of them don't even have a clue about how to use a knife and fork because they have been fed. When the food is fed… rice is mushed with their hand… it doesn't give them that whole experience of the chewing."*

- Health professional

For peer review only

For peer review only - http://bmjopen.bmj.com/site/about/guidelines.xhtml

Theme two: Factors influencing infant feeding and care practices in the British Bangladeshi population

Figure 2: Connecting sub-themes informing theme two: Factors influencing infant feeding and care practices in the British Bangladeshi population

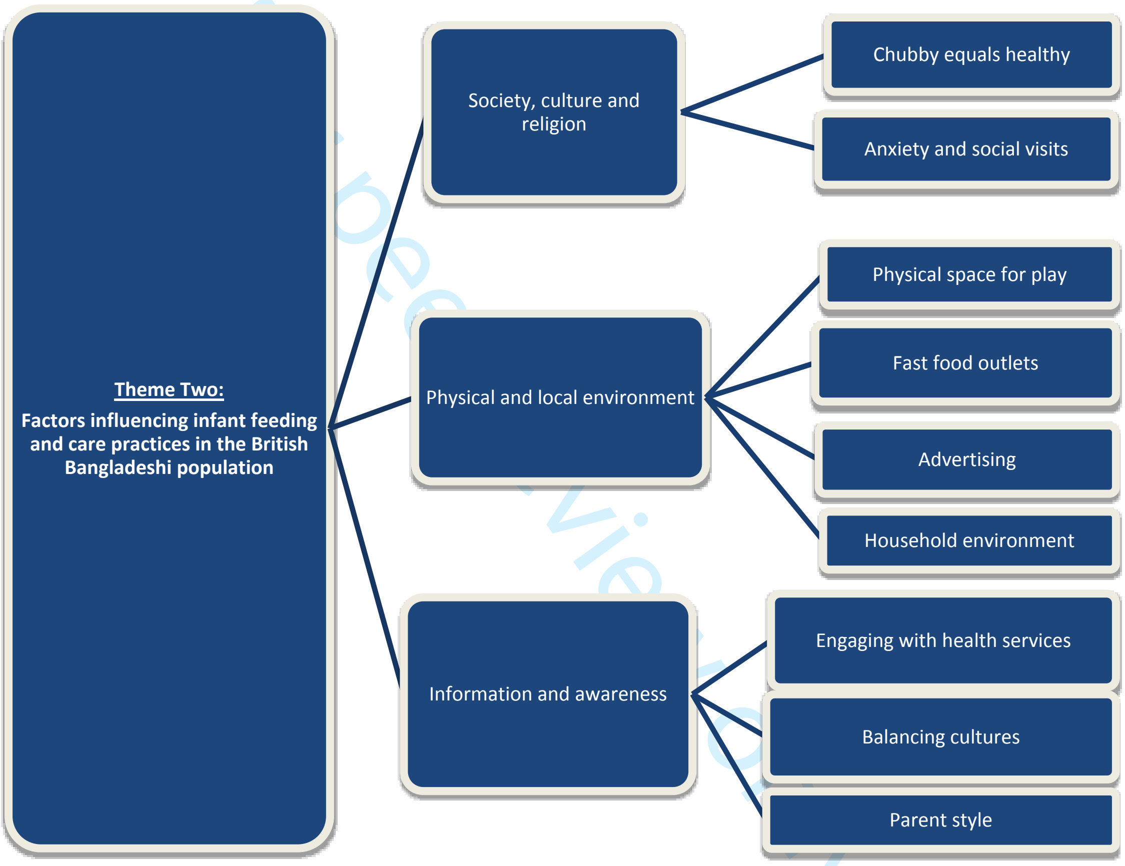

Society, culture and religion

Chubby equals healthy

Community, key informants and parents presented an archetypal societal image of a 'chubby', healthy child. Community members rooted this finding in a belief that extra weight safeguards growth and development during childhood – and this is something that was echoed in the discussions with parents. Parents placed extensive emphasis on good growth and development, which was experienced

by health professionals as a concern for appropriate growth along WHO growth centiles. Health visitors described having guided some mothers to develop a routine to support their meal planning and build resilience against outside community perceptions of a healthy weight. Some parents have learned about the importance of responsive feeding by attending local courses. Community members explained that past generations had experienced a scarcity of resources when raising children in rural Bangladesh and this legacy continues to influence modern feeding practices, although resources are greater and physical activity is lesser in urban East London.

Anxiety and social visits

Mothers reported some anxiety towards social occasions and particularly if they anticipated comments from extended family members or friends', suggesting their child was too skinny, as a critique of not feeding enough, or too chubby, when perceiving jealousy [see quote]. Mothers reported occasionally finding it difficult to challenge social practices, such as offering treats in other people's households, even when they avoided such norms in their own homes.

"If we try to talk to her about [overfeeding her child] she gets offended. In Bengali culture, we have what we call the 'evil eye'.. so, she thinks we are giving her children the 'evil eye' because they eat well, they eat a lot. We still talk to her about it [overfeeding] though, we don't stop."

- Mother

Physical and local environment

Physical space for play

Community members, especially fathers and grandfathers, felt that limited household space left little opportunity for play in some households. The limited physical activity of most family members was often considered to begin in childhood.

For peer review only - http://bmjopen.bmj.com/site/about/guidelines.xhtml

"If you want to be healthy you've got to start from the beginning. Here what do you do? People are living in a box, just in the house or flat, got very limited space for a young child, 9 months or 1-year old child, it's time for them to crawl around. Is there any space to do that? No. Their bone structure, everything, is not getting stronger because they are not exercising, they are not moving around that much. What they do, say put them in a pushchair, in front of the TV and that's it."

- Community member

Elevated levels of outdoor air pollution and concerns for safety in public spaces on rare occasions were added barriers to exercise. Furthermore, exercise was sometimes considered outside of traditional Bangladeshi culture since a historical shift in lifestyles after migration from very physical in rural Bangladesh to more sedentary in the UK. Against this perception, however, some parents encouraged swimming, football or taking walks in the park, facilitated by access to facilities and female-friendly sessions.

Fast food outlets

Community members widely regarded the abundance of fast food outlets in the Tower Hamlets environment as a well-established influence of takeaway meals such as chicken and chips. Some parents reported having a weekly takeaway as a treat. Families also tend to share foods with infants/children and allow them a small taste of meals.

Advertising

Parents found the advertising of semi-solid and solid convenience foods from the age of 4months as confusing and indicative that it is an appropriate age to try solid foods. The availability of formula marketed for 'hungrier babies' supported some parent perceptions that their baby needed more food or more energy dense foods than other babies.

Household environment

The visibility of fruit and vegetables in the household was seen as an easy barrier to overcome in many households. Health professionals and key informants within the community observed that fresh fruit and veg are not usually on

For peer review only - http://bmjopen.bmj.com/site/about/guidelines.xhtml

display in households. Conversely, sweets may be on display in social situations. Health professionals advised helping to introduce different food types and textures by allowing for the visual and touch experience, beyond just taste.

Information and awareness

Engaging with health services

Mothers and Fathers differed in health seeking preferences; fathers stated a preference to speak to a GP whilst mothers described preferring to seek information from sources including Health Visitors, Children's centres, online and community groups. Health professionals and informed community members reported a range of demonstrations and activities for new parents offered at local health and community centres and fundamentally supported with a crèche facility. However, parents and carers widely perceive that advice is constantly changing or conflicting or simply not available. Pregnant women felt antenatal classes would help to explain what to do but not how to feed after birth and fathers sometimes felt ignored by GP's. In general, parents reported wanting more support on topics ranging from: how to introduce solid foods, when to introduce semi/solid foods, when to stop giving milk, what foods to give, in what ratio and how much.

"I am always confused, I am always ringing up the doctors and asking them questions all the time, especially about the milk thing. They said it depends on your child, every child is different. I was a bit like he's still little, doesn't he need the nutrients in the powder to grow?"

- Mother

First time parents especially wished to have more information and support. Health professionals reported that complementary feeding and care needs to become a routine aspect of post-natal care services and that many would benefit from a more consistent '*hand-holding'* approach that is currently lacking.

For peer review only - http://bmjopen.bmj.com/site/about/guidelines.xhtml

Balancing cultures

Mothers often tried to interpret information from different cultures and generations in their infant feeding practices. Grandparents and in-laws were viewed as either supportive influences or pressurising depending on the household. Parents reported adverse feeding practices amongst extended family members, including: feeding extra meals, feeding before bed and placing children in front of the TV.

"By starving, I actually used that term, starving may mean they think the child has not eaten.. like for example if they have not eaten in two or three hours... I will give you a perfect example, I went to the wedding over the weekend and my mother in law was on my case to feed my son... I just said when he is hungry he can communicate with me, he will come up to me and say mum I want to eat. I tried three times, after three times I am not going to go round walking after him. My mother in law you know how she is she is so protective of her grandchildren.. she took him and she fed him, so can you see the difference?"

- Mother

Grandparents reported having usually experienced very different advice and support whilst they were parenting their own children, to parents living in the UK and this appeared to create a generational gap in knowledge, lived experience, awareness and practice that was recognised by both generations. Some grandparents reported feeling like their children did not listen to their advice whilst some mothers and carers reported their mothers or mothers in law provided conflicting advice to that of health professionals. However, these opinions were not universal.

"I remember before I got married before I had my kids they used to do what my mum did which my mum did twenty three years ago, twenty four years ago, at that time it was just egg custard and stuff so they did not know much about vegetables, they did now know much about a lot of stuff. Even when we were growing up

For peer review only - http://bmjopen.bmj.com/site/about/guidelines.xhtml

probably my mum gave like at six months they started introducing what they ate but before that they gave more like egg custards"

- Mother

Some mothers felt they needed to be '*very resilient*' to continue breastfeeding, whilst others felt that grandmothers or mothers in law were supportive of exclusive breastfeeding. Commonly, however, many mothers were proactive in seeking information from different sources, and some mothers felt they were able to resist unhelpful advice by '*being a strong person*' and '*doing my own research and asking advice*', or by having a close network of experienced mums.

"[If] they are fussy eater, it is just because they were fed on only milk because of my daughter's in laws, they are afraid that house will be a mess. They do not know the foods, you have to try foods to know."

- Grandmother

Parent style

Mothers described experiencing anxiety when they felt they did not have enough or the right information and when they felt they did not know how to feed their child, a commonly expressed concern in mothers of 'fussy eaters'.

"Yes, I am worried about him because he's not eating. I try and give him, if he eats anything like the other day I gave him a choc chip roll when I shouldn't have, like it's not good for him to have and he ate it and I was so happy. He never says no to chocolate though, that's a bad thing. Milky bar, his grandparents, always milky bars, he loves it."

- Mother

A more relaxed or confident approach was found amongst mothers that considered themselves as pro-active in seeking information.

For peer review only - http://bmjopen.bmj.com/site/about/guidelines.xhtml

"I notice in our community we have a real thing about feeding them and making sure the fact they are having a full ... do you see what I mean but I just tend to find that be a bit relaxed, see if they take to it, if they eat or whatever. If they don't eat, come back to it, come back to the same meal, come back to that thing. It might be you try something else, they might like it but don't become stressed by it."

- Mother

These mothers recognised the various sources of information and invested time in engaging with different groups, either online or in the community depending on their time, locality and interest. Some mothers considered themselves as a role model to their children and tried to adopt healthy practices to that effect or developed a daily routine with the support of health visitors.

For peer review only - http://bmjopen.bmj.com/site/about/guidelines.xhtml

Figure 3: A framework for priority infant feeding and care practices in British Bangladeshi communities and the contributing factors that need to be addressed under a socio-ecological approach.

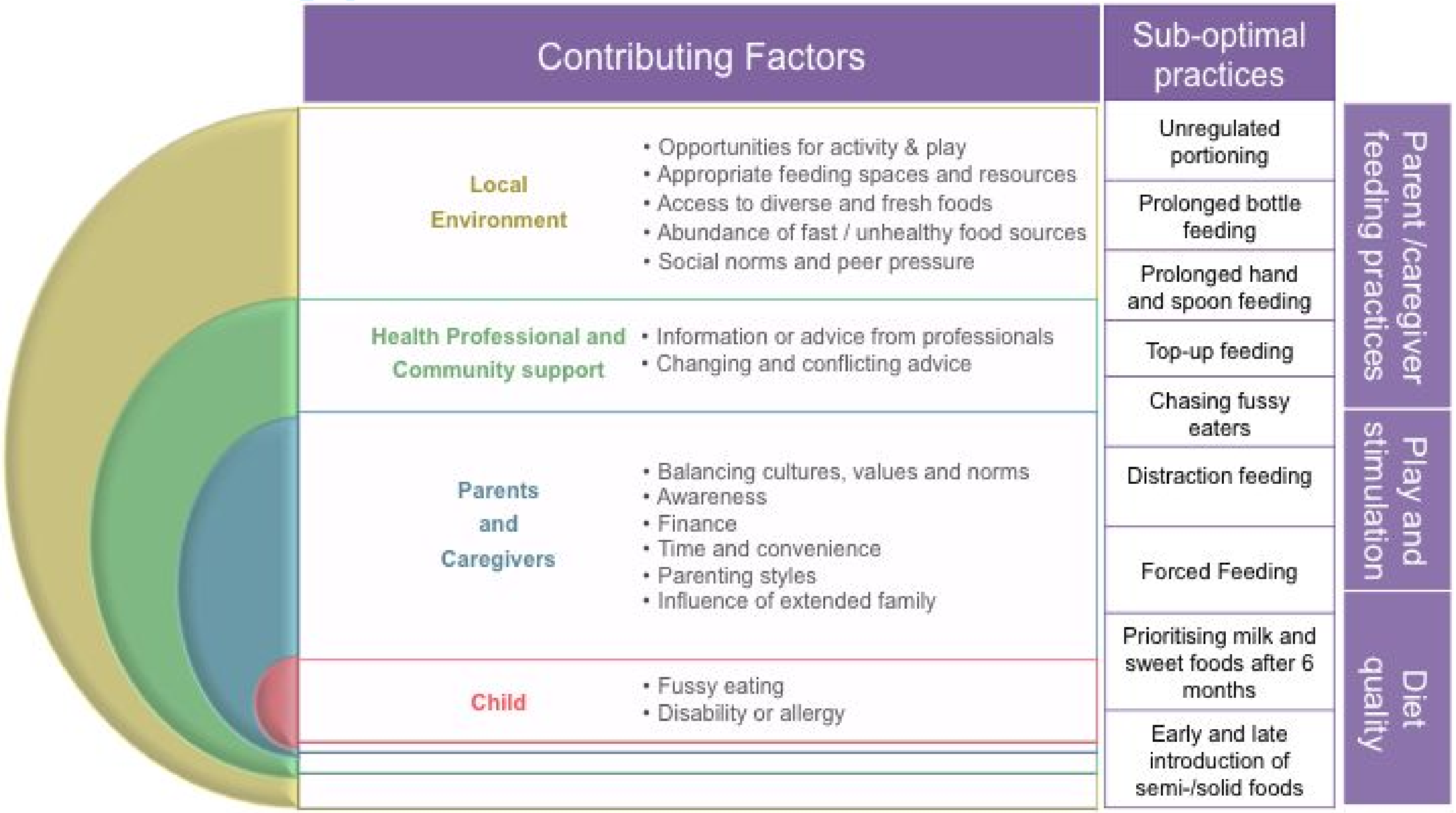

DISCUSSION

Our study was designed to explore complementary infant feeding practices and their drivers within a minority ethnic population of the UK at high risk of suboptimal nutrition and at risk of health problems later in life such as obesity, heart disease, diabetes and dental caries. A unique community partnership and inclusive strategy targeted parents, caregivers, pregnant women, health professionals and the wider Bangladeshi community of Tower Hamlets and provided a diverse range of perspectives and experiences. This extensive range of data was triangulated to provide a reliable, rich and contextualised understanding of the different complementary feeding practices within the British-Bangladeshi population.

Key findings

We identified an assortment of practices that can act to restrict dietary diversity, override infant satiety, facilitate a reliance on sugary or sweetened foods and drink, and encourage over-feeding. Common parent-led feeding practices make it difficult for many parents to pick up on satiety reflexes in the infant/child. Parents often feed when they perceive the child as hungry or to show affection but common practices such as; holding a baby in arms, distracting the child with a phone/TV and prolonged parent- led feeding (via spoon, bottle or hand) can over-ride childhood satiety cues and impact appetite, oral hygiene and speech and language development in childhood and across the life-course.

Figure 3 provides a thematic representation of factors that contribute to sub-optimal infant feeding practices spanning the socio-ecological environment. Of note, the local food environment is a complex system known to shape individual diet [36]. Participants of this study described social, built and natural influencing factors of infant feeding (figure 3). Societal perceptions of 'chubby' as healthy was an especially prominent theme and discussed in tandem with anxiety amongst those that felt they were not following normative approaches to 'filling the belly'. Challenges extend to the local environment, where participants identified a lack of opportunities for play and physical activity, appropriate

For peer review only

feeding spaces, an abundance of fast food outlets and misleading advertisements of infant foods. Participants also emphasised the benefits of access to children centres and health visitors in Tower Hamlets, for accessible support and problem solving.

Consistency

Our findings are consistent with evidence of an association between ethnicity, infant feeding practices and BMI amongst the British and South Asian populations in Bradford [37]. We add to emerging evidence from Tower Hamlets that identifies a myriad of factors, including family and friends as influencers of infant feeding practices in Tower Hamlets [38] and a systematic review that recognises the value of heritage, tradition and culture on infant feeding practices within South Asian families living in high income settings [28].

Strengths and limitations

Culturally acceptable interventions to promote optimal infant feeding and care amongst minority populations have been relatively under-researched to date. Existing literature does not adequately capture the cultural and social determinants of infant feeding and care. A strength of this study is the development of culturally sensitive interview tools and recruitment strategies, based on evidence from the literature and guided by our PPI and community facilitators. This strong partnership with members of the British-Bangladeshi population of Tower Hamlets enabled further reach and diversity of representation in this study. A limitation of this qualitative study is that a quantitative measure of dietary intake was out of scope. However, cohort studies explore the association between infant dietary intake and ethnicity [39]. Another limitation is that all data are self-reported and have the potential for respondent bias. We believe we overcame this limitation through triangulation of respondent data, a phased approach that encouraged further probing into emerging topics between stakeholder groups and by collecting data from a relatively large sample.

For peer review only

Implications for clinicians and policymakers

These qualitative data add strength to the debate around regulation and marketing of follow on infant formula and foods. In 2016, the UK launched 'Childhood obesity: a plan for action (2016)', in which the Department of Health set the target to reduce England's rate of childhood obesity within the next 10 years. The action plan focuses on partnership with the private sector but has been criticised for an inadequate stance on regulatory measures [40]. The sugar tax was a celebrated feature of the plan, but comparatively little political action has been taken towards early childhood nutrition or enforcing advertising regulations. Updated SACN guidelines emphasise educating parents and caregivers on behavioural practices relating to infant feeding, including introducing new foods, textures and diversity, yet this will take time to reach households. As such, the current model of early feeding support can be perceived as absent, untimely or confusing for caregivers.

More regular contact between pregnancy and 24months, signposting to existing and local resources and additional support in the form of facilitator led PLA women's groups could improve the current model of infant feeding support. The economic incentive alone for early nutritional intervention in the UK is profound; treating obesity-related disease is estimated to cost the National Health Service (NHS) £6.1 billion pounds per year and the wider costs to society estimated at around three times this [41]. A growing interest in reverse innovation [42] –the concept of transferring a product or service developed in a resource poor setting to an industrialised setting [43] - has led to demonstrable cost savings for the NHS in areas from technology [44] to community mobilisation [45]. There remains a question of who and how to deliver effective community-based programmes under the NHS? Local leadership is essential; this study was fostered in part by a supportive local agenda, where the Health and Well-Being Board enshrine 'Children's Weight and Nutrition' as one of five top priorities. Similar boards provide a comparable opportunity for action and partnership with civil society and private sector if sustained by an ideology rooted in the social determinants of health.

For peer review only - http://bmjopen.bmj.com/site/about/guidelines.xhtml

Transferability

This study provides an in-depth understanding of cultural and social influencers of infant feeding and care within the largest national British Bangladeshi population and supports observations from other South Asian populations in the UK [37,39]. These findings may be transferable to British Bangladesh residents across the UK as well as South Asian populations and other ethnic minority groups. A broad range of stakeholders, including health professionals, informed our understanding of the shared environmental challenges to infant feeding and care and need for improved Infant and Young Child Feeding services (IYCF) that exist nationally. We recommend that this tailored approach to exploring IYCF practices be extended to other ethnic minority populations in the UK. Finally, while this study focuses on an ethnic subgroup, the dual burden of malnutrition is a global concern. Data on IYCF indicators are limited but the data that are available show concerns for complementary feeding practises across high, middle and low-income settings that are in line with our study findings. We suggest that while many themes may be generalizable, culturally tailored approaches will be required to explore IYCF in other settings. We seek to use this formative research to inform the adaptation of a participatory and community-based intervention in an NHS context, which could have far -reaching implications for programme design and vulnerable populations in high-income settings.

Future research

This study identifies the need for more evidence to inform services that effectively optimise feeding and care in the early years and target vulnerable populations in the UK. We recommend for more research to support the development of evidence-based interventions that optimise feeding practices across all ethnic majority and minority populations in the UK under a community based participatory approach. We encourage culturally sensitive and community-based programming that facilitates families to discuss and challenge societal norms underpinning infant feeding practices, considerate of the whole family unit, including fathers and grandparents. More research is needed to inform an integrated participatory approach so that caregivers and community

For peer review only - http://bmjopen.bmj.com/site/about/guidelines.xhtml

can feasibly be included in an NHS-community partnership to co-develop infant and young child feeding services. As the PLA group approach has been demonstrated to bring statistically significant improvements in maternal and infant survival through application with participatory women's groups in low and middle-income settings [24]; NEON, therefore, ultimately seeks to determine whether an adapted PLA group approach has the potential for early and effective intervention, using infant feeding as an exemplar.

Conclusion

We recommend that effective, culturally sensitive support be provided to parents, caregivers and extended family members at different 'ages and stages' through evidence based and tailored infant feeding programmes.

For peer review only

Acknowledgments

The authors would like to thank the National Institute of Health Research, Collaboration for Leadership in Applied Health Research and Care North Thames for funding the NEON study.

We would like to thank all NEON study team members for their contribution and guidance to research activities throughout the three phases of the NEON study. All study team members made considerable contribution to research activities and decision making throughout duration of the study and supported identification of key study findings and recommendations stated in this report. We would like to thank the NEON study implementation partners, who supported and participated in research activities and enabled the research process to proceed effectively. NEON study implementation partners included Tower Hamlets Borough Local Authority, Barts Health NHS Trust, Newham Local Authority, Women and Children First, Women's Health and Family Services, and The Breastfeeding Network in Tower Hamlets. Thanks go to the NEON advisory board, made up researchers, practitioners, study partners and patient public involvement members who attended NEON advisory meetings, offered guidance and supported decision making on study activities. We would like to thank the NEON study's community and Participatory Learning and Action group facilitators who performed integral roles in data collection and NEON intervention delivery activities. Finally, we would also like to thank the British Bangladeshi population in Tower Hamlets for participating in and supporting the research.

Competing interest statement:

"All authors have declared that no support from any organisation for the submitted work; no financial relationships with any organisations that might have an interest in the submitted work in the previous three years, no other relationships or activities that could appear to have influenced the submitted work."

For peer review only - http://bmjopen.bmj.com/site/about/guidelines.xhtml

Ethical approval: The project was approved for research ethics from the University College London Research Ethics Committee (Ethics project ID number: 10271001)

Funding: This work was supported by National Institute of Health Research, CLAHRC / Barts Health

For peer review only

References

1. Schenker S: Briefing paper: Undernutrition in the UK. British Nutrition Foundation Nutrition Bulletin 2003, 28:87-120.
2. Butland, Bryony, et al. "Foresight. Tackling obesities: future choices. Project report." *Foresight. Tackling obesities: future choices. Project report.* (2007).
3. NHS Digital National Child Measurement Programme. (Jan 14, 2019). https://digital.nhs.uk/areas-of-interest/public-health/national-child-measurement-programme Date: 2006
4. WHO: http://www.euro.who.int/en/health-topics/noncommunicable-diseases/obesity/data-and-statistics).
5. Bammann, Karin, et al. "Early life course risk factors for childhood obesity: the IDEFICS case-control study." *PloS one*9.2 (2014): e86914.
6. Roseboom, Tessa J., et al. "Coronary heart disease after prenatal exposure to the Dutch famine, 1944–45." *Heart* 84.6 (2000): 595-598.Roseboom T, de Rooij S, Painter R. Early Hum Dev 2006;82:485-491.
7. Eriksson, Johan G. "Epidemiology, genes and the environment: lessons learned from the Helsinki Birth Cohort Study." *Journal of internal medicine* 261.5 (2007): 418-425.
8. Prescott, Susan, and Richard Saffery. "The role of epigenetic dysregulation in the epidemic of allergic disease." *Clinical epigenetics* 2.2 (2011): 223.
9. Langley-Evans, S. C. "Nutrition in early life and the programming of adult disease: a review." *Journal of Human Nutrition and Dietetics* 28 (2015): 1-14.
10. Lawlor, Debbie A., et al. "Associations of birth size and duration of breast feeding with cardiorespiratory fitness in childhood: findings from the Avon Longitudinal Study of Parents and Children (ALSPAC)." *European journal of epidemiology* 23.6 (2008): 411-422.
11. Gaillard, Romy, et al. "Childhood cardiometabolic outcomes of maternal obesity during pregnancy: the Generation R Study." *Hypertension* 63.4 (2014): 683-691.
12. Rechel, Bernd, et al. "The role of public health organizations in addressing public health problems in Europe." (2018).
13. Magnusson, Maria, et al. "Social inequalities in obesity persist in the Nordic region despite its relative affluence and equity." *Current obesity reports* 3.1 (2014): 1-15.
14. Mughal Z: Resurgence of vitamin D deficiency rickets in UK. Endocrine Abstracts 2012, 28 S7.4.
15. Lawson MS, Thomas M, Hardiman A: Iron status of Asian children aged 2 years living in England. Arch Dis Child 1998, 78(5):420-426.
16. National Obesity Observatory, 2013. Public Health England. National Child Measurement Programme (NCMP): Changes in children's BMI between 2006/07 and 2011/12, p 18. In.
17. Marcenes, W., et al. "Ethnic disparities in the oral health of three-to four-year-old children in East London." *British dental journal* 215.2 (2013): E4.
18. Perez-Escamilla, Rafael, et al. "Nutrition disparities and the global burden of malnutrition." *BMJ* 361 (2018): k2252. (https://www.bmj.com/content/361/bmj.k2252)

For peer review only - http://bmjopen.bmj.com/site/about/guidelines.xhtml

19. Feeding in the First Year of Life. SACN Report. 2018 (https://assets.publishing.service.gov.uk/government/uploads/system/uploads/attachment_data/file/725530/SACN_report_on_Feeding_in_the_First_Year_of_Life.pdf)
20. Daniels, Lynne A., et al. "The NOURISH randomised control trial: positive feeding practices and food preferences in early childhood-a primary prevention program for childhood obesity." BMC public health 9.1 (2009): 387.
21. Florin, Dominique, and Jennifer Dixon. "Public involvement in health care." BMJ: British Medical Journal 328.7432 (2004): 159.
22. Department of Health. "Liberating the NHS: No decision about me, without me." (2012).
23. Crisp, Nigel. "Mutual learning and reverse innovation–where next?." *Globalization and health* 10.1 (2014): 14.
24. Seward, Nadine, et al. "Effects of women's groups practising participatory learning and action on preventive and care-seeking behaviours to reduce neonatal mortality: A meta-analysis of cluster-randomised trials." *PLoS medicine* 14.12 (2017): e1002467.
25. Sikorski, Catherine, et al. "Could Postnatal Women's Groups Be Used to Improve Outcomes for Mothers and Children in High-Income Countries? A Systematic Review." *Maternal and child health journal* (2018): 1-15.
26. A systematic review of complementary feeding practices in South Asian infants and young children: the Bangladesh perspective L Manikam, A Robinson, JY Kuah, HJ Vaidya, EC Alexander, GW Miller, BMC Nutrition 3 (1), 56
27. Systematic review of infant and young child complementary feeding practices in South Asian families: the India perspective L Manikam, A Prasad, A Dharmaratnam, C Moen, A Robinson, A Light, Public health nutrition 21 (4), 637-654
28. Manikam L, Lingam R, Lever I, Alexander EC, Amadi C, Milner Y, Shafi T, Stephenson L, Ahmed S, Lakhanpaul M. Complementary Feeding Practices for South Asian Young Children Living in High-Income Countries: A Systematic Review. 2018 November; 10(11)
29. Manikam L, Prasad A, Dharmaratnam A, Moen C, Robinson A, Light A, Ahmed S, Lingam R and Lakhanpaul M. Systematic review of infant and young child complementary feeding practices in South Asian families: the Pakistan perspective. Public Health Nutrition. 2018 March; 21(4):655-668.
30. Lakhanpaul M, Bird D, Culley L, et al. The use of a collaborative structured methodology for the development of a multifaceted intervention programme for the management of asthma (the MIA project), tailored to the needs of children and families of South Asian origin: a community-based, participatory study. Southampton (UK): NIHR Journals Library; 2014 Sep. (Health Services and Delivery Research, No. 2.28.) Available from: http://www.ncbi.nlm.nih.gov/books/NBK260076/.
31. Minkler, Meredith. "Community-based research partnerships: challenges and opportunities." *Journal of Urban Health* 82.2 (2005): ii3-ii12.

For peer review only - http://bmjopen.bmj.com/site/about/guidelines.xhtml

32. London’s Poverty Profile 2017, Trust for London and New Policy Institute https://www.trustforlondon.org.uk/publications/londons-poverty-profile-2017/
33. Choudhry, Kubra, and Louise M. Wallace. "‘Breast is not always best’: South Asian women's experiences of infant feeding in the UK within an acculturation framework." *Maternal & child nutrition* 8.1 (2012): 72-87.
34. Braun, Virginia, et al. "Thematic analysis." *Handbook of Research Methods in Health Social Sciences* (2019): 843-860.
35. Edlund, B., and A. McDougall. "NVivo 11 Essentials. Your guide to the leading qualitative Analysis Software." (2016).
36. Engler-Stringer, Rachel, et al. "The community and consumer food environment and children’s diet: a systematic review." *BMC public health* 14.1 (2014): 522.
37. Santorelli, Gillian, et al. "Ethnic differences in infant feeding practices and their relationship with BMI at 3 years of age–results from the Born in Bradford birth cohort study." British Journal of Nutrition 111.10 (2014): 1891-1897.
38. Rayment, Juliet, et al. "Bangladeshi women's experiences of infant feeding in the London Borough of Tower Hamlets." Maternal & child nutrition 12.3 (2016): 484-499.
39. Sahota, Pinki, et al. "Ethnic differences in dietary intake at age 12 and 18 months: the Born in Bradford 1000 Study." Public health nutrition 19.1 (2016): 114-122.
40. Country report for England, see Online Appendix.
41. Public Health England, 2017: Public Health England (2018). Public Health Profiles [website]. London: Public Health England (https://fingertips.phe.org.uk/, accessed 8 June 2018).
42. Crisp, Nigel. "Co-development, innovation and mutual learning–or how we need to turn the world upside down." *Healthcare*. Vol. 3. No. 4. Elsevier, 2015.
43. Bhatti, Y. A. "Reverse innovation: Create far from home, win everywhere." *South Asian Journal of Global Business Research* 3.1 (2014): 102-104.
44. Prime, Matthew, et al. "From Malawi to Middlesex: the case of the Arbutus Drill Cover System as an example of the cost-saving potential of frugal innovations for the UK NHS." *BMJ Innovations* 4.2 (2018): 103-110.
45. Johnson, Christopher David, et al. "Learning from the Brazilian community health worker model in North Wales." *Globalization and health* 9.1 (2013): 25.

For peer review only - http://bmjopen.bmj.com/site/about/guidelines.xhtml

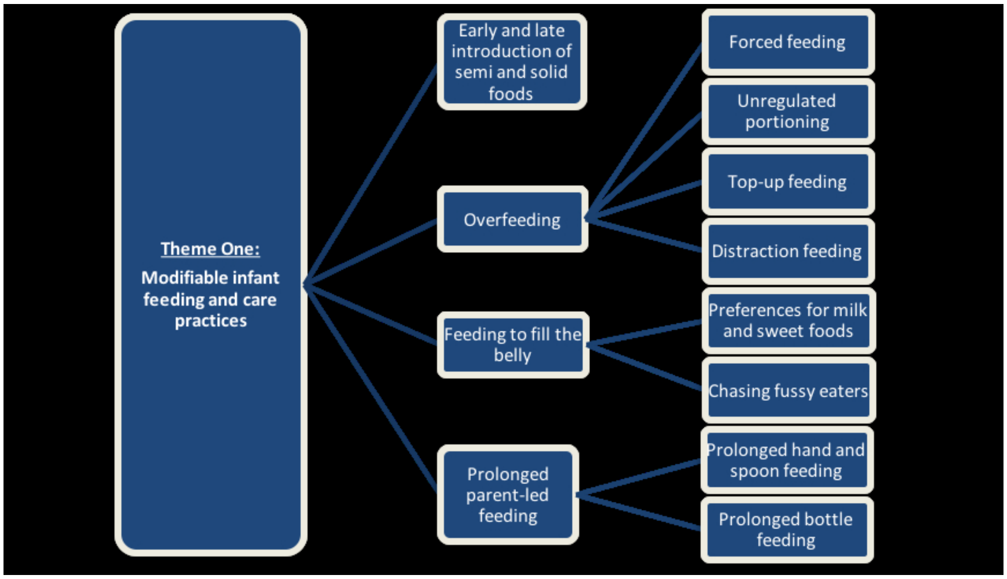
Theme One:
Modifiable infant feeding and care practices
Early and late introduction of semi and solid foods
Overfeeding
Feeding to fill the belly
Prolonged parent-led feeding
Forced feeding
Unregulated portioning
Top-up feeding
Distraction feeding
Preferences for milk and sweet foods
Chasing fussy eaters
Prolonged hand and spoon feeding
Prolonged bottle feeding

Figure 1

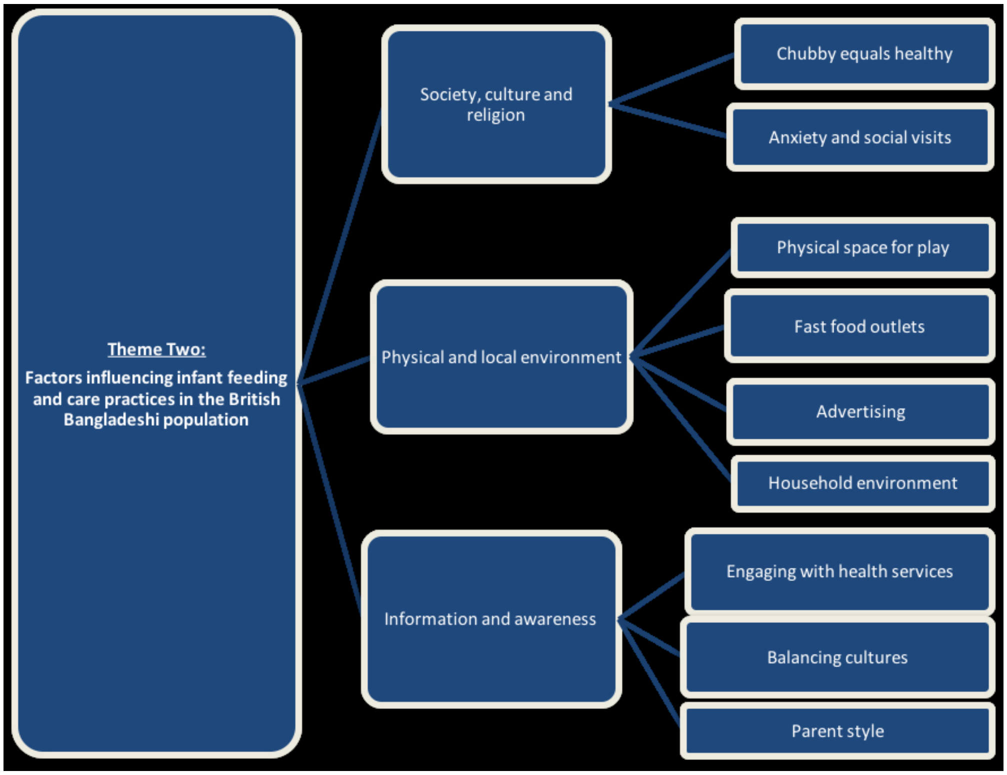
Theme Two:
Factors influencing infant feeding and care practices in the British Bangladeshi population
Society, culture and religion
Chubby equals healthy
Anxiety and social visits
Physical and local environment
Physical space for play
Fast food outlets
Advertising
Household environment
Information and awareness
Engaging with health services
Balancing cultures
Parent style

Figure 2

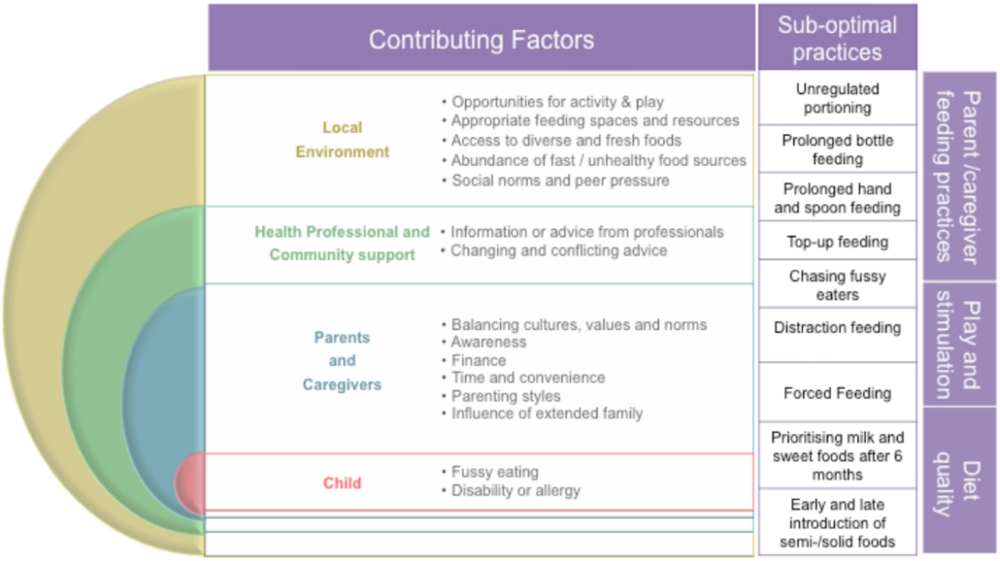
Contributing Factors
Sub-optimal practices
Local Environment
• Opportunities for activity & play
• Appropriate feeding spaces and resources
• Access to diverse and fresh foods
• Abundance of fast / unhealthy food sources
• Social norms and peer pressure
Health Professional and Community support
• Information or advice from professionals
• Changing and conflicting advice
Parents and Caregivers
• Balancing cultures, values and norms
• Awareness
• Finance
• Time and convenience
• Parenting styles
• Influence of extended family
Child
• Fussy eating
• Disability or allergy
Unregulated portioning
Prolonged bottle feeding
Prolonged hand and spoon feeding
Top-up feeding
Chasing fussy eaters
Distraction feeding
Forced Feeding
Prioritising milk and sweet foods after 6 months
Early and late introduction of semi-/solid foods
Parent /caregiver feeding practices
Play and stimulation
Diet quality

Figure 3

Reporting checklist for qualitative study.

Based on the SRQR guidelines.

Instructions to authors

Complete this checklist by entering the page numbers from your manuscript where readers will find each of the items listed below.

Your article may not currently address all the items on the checklist. Please modify your text to include the missing information. If you are certain that an item does not apply, please write "n/a" and provide a short explanation.

Upload your completed checklist as an extra file when you submit to a journal.

In your methods section, say that you used the SRQRreporting guidelines, and cite them as:

O'Brien BC, Harris IB, Beckman TJ, Reed DA, Cook DA. Standards for reporting qualitative research: a synthesis of recommendations. Acad Med. 2014;89(9):1245-1251.

		Reporting Item	Page Number
**Title**			
	#1	Concise description of the nature and topic of the study identifying the study as qualitative or indicating the approach (e.g. ethnography, grounded theory) or data collection methods (e.g. interview, focus group) is recommended	1,3
**Abstract**			
	#2	Summary of the key elements of the study using the abstract format of the intended publication; typically includes background, purpose, methods, results and conclusions	3
**Introduction**			
Problem formulation	#3	Description and signifcance of the problem / phenomenon studied: review of relevant theory and empirical work; problem statement	5,6
Purpose or research question	#4	Purpose of the study and specific objectives or questions	6,7

For peer review only

Methods

Qualitative approach and research paradigm	#5	Qualitative approach (e.g. ethnography, grounded theory, case study, phenomenolgy, narrative research) and guiding theory if appropriate; identifying the research paradigm (e.g. postpositivist, constructivist / interpretivist) is also recommended; rationale. The rationale should briefly discuss the justification for choosing that theory, approach, method or technique rather than other options available; the assumptions and limitations implicit in those choices and how those choices influence study conclusions and transferability. As appropriate the rationale for several items might be discussed together.	7
Researcher characteristics and reflexivity	#6	Researchers' characteristics that may influence the research, including personal attributes, qualifications / experience, relationship with participants, assumptions and / or presuppositions; potential or actual interaction between researchers' characteristics and the research questions, approach, methods, results and / or transferability	7,8
Context	#7	Setting / site and salient contextual factors; rationale	8
Sampling strategy	#8	How and why research participants, documents, or events were selected; criteria for deciding when no further sampling was necessary (e.g. sampling saturation); rationale	8,9
Ethical issues pertaining to human subjects	#9	Documentation of approval by an appropriate ethics review board and participant consent, or explanation for lack thereof; other confidentiality and data security issues	10,11
Data collection methods	#10	Types of data collected; details of data collection procedures including (as appropriate) start and stop dates of data collection and analysis, iterative process, triangulation of sources / methods, and modification of procedures in response to evolving study findings; rationale	8,9
Data collection instruments and technologies	#11	Description of instruments (e.g. interview guides, questionnaires) and devices (e.g. audio recorders) used for data collection; if / how the instruments(s) changed over the course of the study	10
Units of study	#12	Number and relevant characteristics of participants, documents, or events included in the study; level of participation (could be	12

For peer review only

		reported in results)	
Data processing	#13	Methods for processing data prior to and during analysis, including transcription, data entry, data management and security, verification of data integrity, data coding, and anonymisation / deidentification of excerpts	10
Data analysis	#14	Process by which inferences, themes, etc. were identified and developed, including the researchers involved in data analysis; usually references a specific paradigm or approach; rationale	11
Techniques to enhance trustworthiness	#15	Techniques to enhance trustworthiness and credibility of data analysis (e.g. member checking, audit trail, triangulation); rationale	7,8
**Results/findings**			
Syntheses and interpretation	#16	Main findings (e.g. interpretations, inferences, and themes); might include development of a theory or model, or integration with prior research or theory	12-29
Links to empirical data	#17	Evidence (e.g. quotes, field notes, text excerpts, photographs) to substantiate analytic findings	14-29
**Discussion**			
Intergration with prior work, implications, transferability and contribution(s) to the field	#18	Short summary of main findings; explanation of how findings and conclusions connect to, support, elaborate on, or challenge conclusions of earlier scholarship; discussion of scope of application / generalizability; identification of unique contributions(s) to scholarship in a discipline or field	31-35
Limitations	#19	Trustworthiness and limitations of findings	32
**Other**			
Conflicts of interest	#20	Potential sources of influence of perceived influence on study conduct and conclusions; how these were managed	36
Funding	#21	Sources of funding and other support; role of funders in data collection, interpretation and reporting	7, 36

None The SRQR checklist is distributed with permission of Wolters Kluwer © 2014 by the Association of American Medical Colleges. This checklist can be completed online using https://www.goodreports.org/, a tool made by the EQUATOR Network in collaboration with Penelope.ai

For peer review only

BMJ Open

Nurture Early for Optimal Nutrition (NEON) Programme: Qualitative study of drivers of infant feeding and care practices in a British-Bangladeshi population

Journal:	*BMJ Open*
Manuscript ID	bmjopen-2019-035347.R1
Article Type:	Original research
Date Submitted by the Author:	14-Apr-2020
Complete List of Authors:	Lakhanpaul, Monica; University College London Institute of Child Health, Population, Policy and Practice; Whittington Health NHS Trust<br>Benton, Lorna; University College London Institute of Child Health, Population, Policy and Practice<br>Lloyd-Houldey, Oliver; University College London Institute of Child Health, Population, Policy and practice<br>Manikam, Logan; University College London Institute of Epidemiology and Health Care, Department of Epidemiology and Public Health; Aceso Global Health Consultants Ltd<br>Rosenthal, Diana; University College London Institute of Child Health, Population, Policy and Practice<br>Allaham, Shereen ; University College London Institute of Epidemiology and Health Care, Department of Epidemiology and Public Health; Aceso Global Health Consultants Ltd<br>Heys, Michelle ; University College London Institute of Child Health, Population, Policy and Practice
**Primary Subject Heading**:	Paediatrics
Secondary Subject Heading:	Nutrition and metabolism, Public health
Keywords:	Community child health < PAEDIATRICS, NUTRITION & DIETETICS, PUBLIC HEALTH

SCHOLARONE™
Manuscripts

BMJ

I, the Submitting Author has the right to grant and does grant on behalf of all authors of the Work (as defined in the below author licence), an exclusive licence and/or a non-exclusive licence for contributions from authors who are: i) UK Crown employees; ii) where BMJ has agreed a CC-BY licence shall apply, and/or iii) in accordance with the terms applicable for US Federal Government officers or employees acting as part of their official duties; on a worldwide, perpetual, irrevocable, royalty-free basis to BMJ Publishing Group Ltd ("BMJ") its licensees and where the relevant Journal is co-owned by BMJ to the co-owners of the Journal, to publish the Work in this journal and any other BMJ products and to exploit all rights, as set out in our licence.

The Submitting Author accepts and understands that any supply made under these terms is made by BMJ to the Submitting Author unless you are acting as an employee on behalf of your employer or a postgraduate student of an affiliated institution which is paying any applicable article publishing charge ("APC") for Open Access articles. Where the Submitting Author wishes to make the Work available on an Open Access basis (and intends to pay the relevant APC), the terms of reuse of such Open Access shall be governed by a Creative Commons licence – details of these licences and which Creative Commons licence will apply to this Work are set out in our licence referred to above.

Other than as permitted in any relevant BMJ Author's Self Archiving Policies, I confirm this Work has not been accepted for publication elsewhere, is not being considered for publication elsewhere and does not duplicate material already published. I confirm all authors consent to publication of this Work and authorise the granting of this licence.

For peer review only

Title

Nurture Early for Optimal Nutrition (NEON) Programme: Qualitative study of drivers of infant feeding and care practices in a British-Bangladeshi population

Authors and affiliation

1. Monica Lakhanpaul

Population, Policy and Practice Research and Teaching Department, University College London Institute of Child Health, London, UK

Whittington Health NHS Trust, London, UK, N19 5NF

Lorna Benton

Population, Policy and Practice Research and Teaching Department, University College London Institute of Child Health, London, UK

2. Oliver Lloyd-Houldey

Population, Policy and Practice Research and Teaching Department, University College London Institute of Child Health, London, UK

3. Logan Manikam

Department of Epidemiology and Public Health, University College London Institute of Epidemiology and Health Care, London, UK

Aceso Global Health Consultants Ltd, London, UK

4. Diana Margot Rosenthal

Population, Policy and Practice Research and Teaching Department, University College London Institute of Child Health, London, UK

5. Shereen Allaham

Department of Epidemiology and Public Health, University College London Institute of Epidemiology and Health Care, London, UK

Aceso Global Health Consultants Ltd, London, UK

For peer review only - http://bmjopen.bmj.com/site/about/guidelines.xhtml

6. Michelle Heys

Population, Policy and Practice Research and Teaching Department, University College London Institute of Child Health, London, UK

Corresponding author:

Monica Lakhanpaul

Professor of Integrated Community Child Health, UCL Great Ormond Street Institute of Child Health, London WC1N 1EH.

E-mail: m.lakhanpaul@ucl.ac.uk

Telephone number: +442079052259

Word Count: 9,172 (ex title page, abstract, references, figures and tables)

For peer review only - http://bmjopen.bmj.com/site/about/guidelines.xhtml

Abstract

Objectives: To explore optimal infant feeding and care practices and their drivers within the British-Bangladeshi population of East London, UK as an exemplar to inform development of a tailored, co-adapted participatory community intervention.

Design: Qualitative community-based participatory research

Setting: Community and children's centres and NHS settings within Tower Hamlets, London, UK

Participants: 141 participants completed the community study including: British-Bangladeshi mothers, fathers, grandmothers and grandfathers of infants and young children aged 6-23months, key informants and lay community members from the British-Bangladeshi population of Tower Hamlets, and health professionals working in Tower Hamlets.

Results: 141 participants from all settings and generations identified several infant feeding and care practices and wider socio-ecological factors that could be targeted to optimise nutritional outcomes. Our modifiable infant feeding and care practices were highlighted: untimely introduction of semi- and solid foods, overfeeding, prolonged parent-led feeding and feeding to 'fill the belly'. Wider socio-ecological determinants were highlighted, categorised here as: (1) society and culture (for example, equating "chubby baby" to healthy baby), (2) physical and local environment (e.g. fast food outlets, advertising), and (3) information and awareness (for example, communication with healthcare professionals around cultural norms).

Conclusions: Parenting interventions should be co-developed with communities and tailored to recognise and take account of social and cultural norms and influence from different generations that inform infant feeding and care practices and may be of particular importance for infants from ethnically diverse communities. In addition, UK infant feeding environment requires better regulation of marketing of foods for infants and young children if it is to optimise nutrition in the early years.

For peer review only - http://bmjopen.bmj.com/site/about/guidelines.xhtml

Keywords: British Bangladeshi; Nutrition; Children; Infant; Feeding Practice; Early Interventions; Complementary Feeding; participatory; community

Strengths and limitations of this study

- Existing literature does not adequately describe the cultural and social determinants of infant feeding and care practices. This study addresses this gap by generating transferable lessons from the British- Bangladeshi population to inform similar studies to inform co-developed, adapted or tailored programmes sensitive to the needs of culturally diverse communities.

- A strength of this study is the development of culturally and generational sensitive interview tools and recruitment strategies. The methodology for the development and the tools/strategies themselves can be generalised to other populations.

- A further strength of the study that the data collected directly informed the wider NEON (Nurture Early for Optimal Nutrition) intervention led by the same study group thereby ensuring rapid transfer of results into a public health intervention.

- The strong partnership developed between community members from the British-Bangladeshi population of Tower Hamlets, London, UK enabled a wider reach and diversity of representation in this study. The study engaged not only with mothers but also members from the wider community and across generations.

- One limitation of this study and potential for future research, is that the qualitative data were not triangulated with a quantitative measure of dietary intake (out of scope for this study). Another limitation is that all data are self-reported and have the potential for respondent bias. However, we report data from a large sample with thematic saturation.

For peer review only - http://bmjopen.bmj.com/site/about/guidelines.xhtml

For peer review only

Nurture Early for Optimal Nutrition (NEON) Programme: Qualitative study of drivers of infant feeding and care practices in a British-Bangladeshi population

INTRODUCTION

The first 1,000 days of life presents a critical window, but also an opportunity to prevent the dual burden of under- and over-nutrition. In the UK, an estimated 5% of the population is undernourished, of which 70% goes unreported [1]. Meanwhile, obesity prevalence continues to rise in accordance with projections that over half of UK adults could be obese by 2050 [2]. Currently, approximately one third of children leave primary school as obese or overweight in the UK [3]. Obesity, alongside potential mediating and wider socio-economic determinants, has both direct and indirect substantial implications for: underachievement in school, lower self-esteem and risk of developing cardiovascular disease or type 2 diabetes into adulthood [4].

Health inequities contribute to the risk of nutritionally-related diseases across the life course, such as diabetes, coronary heart disease and allergies [5-11]. Prevalence of childhood obesity is twice as high amongst those living in the most deprived, compared to the least deprived areas of the UK [12] and a similar trend is observed elsewhere in Europe [13]. Furthermore, acculturating to a host country has been shown to have significant impact on obesogenic behaviours. Migrants from low- and middle-income countries moving to high-income countries tend to be healthier than their local counterparts in the host country when they newly arrived, known as 'healthy migrant effect'. However, immigrants post migration have been found to have poor health and more susceptible to be obese or overweight compared to the host population [14]. Some of the highest burdens of inequity and obesity are measured amongst ethnic minority populations, however a tendency to aggregate data from South Asian (SA) populations limits a full understanding of trends that may result from different sociocultural norms and practices. Previous studies specifically examined nutritional outcomes in the British-Bangladeshi population reporting an increased risk of vitamin D deficiency [15] and iron deficiency [16], which are essential nutrients for brain development in children; the highest proportion of obesity (31%) of any ethnic group amongst 6-year-old boys [17]; and poorer oral health in pre-school children [18].

For peer review only - http://bmjopen.bmj.com/site/about/guidelines.xhtml

The first 1000 days of life have been considered a window of opportunity [19]. Interventions delivered in the early years, and more specifically during the first 1000 days, to optimise nutrition and feeding practices could provide a holistic and potentially cost-effective approach to the prevention of nutritionally-related diseases [18]. Extensive literature exists on barriers to exclusive breastfeeding, yet comparably little attention has been offered to complementary feeding and care practices. The 6-23-month age period is known as the 'weaning' or 'complementary feeding' age, when solid and semi-solid foods are nutritionally required to support breastfeeding. In 2018, the Scientific Advisory Committee on Nutrition (SACN) published the much-anticipated 'Feeding in the First Year of Life' report. Although many recommendations remained unchanged, it recognised the importance of feeding practices and caregiver behaviours such as familiarising infants to a range of flavours and textures [20].

One forthcoming challenge is how to translate SACN guidelines into effective infant feeding programmes. Evidence demonstrates that culturally sensitive infant and young child feeding (IYCF) programmes need to be tailored to the target population and inclusive of the extended family and friends that typically offer more regular and often traditional support [21]. Although the National Health Service (NHS) has long recognised the value of community-centred approaches, few studies explore cultural and social drivers of complementary feeding practices to inform models that utilise local resources and assets [22,23]. There is an opportunity to learn from low-resource approaches that have proven to be effective globally [24-26] and build local partnerships to develop tailored community-based approaches. The results in this paper represent the formative phase of a wider programme of work (NEON- Nurture Early for Optimal Nutrition) [27]. NEON 1 initially engaged with British-Bangladeshi communities to develop generalisable evidence and an adapted Participatory Learning and Action group approach. This was subsequently tested for its acceptability in the community and to identify any refinements that may be required in the approach (delivery and content of the intervention). The approach will be followed by NEON 2 where it will be delivered to other SA communities with the aim, if successful to potentially other diverse minority ethnic populations in the future to ensure a more inclusive approach to health promotion interventions.

For peer review only - http://bmjopen.bmj.com/site/about/guidelines.xhtml

METHODOLOGY

Study design

The NEON 1 (Nurture Early for Optimal Nutrition) is a National Institute for Health Research funded study that aims to explore common complementary feeding and care practices and their social and cultural influences within the British-Bangladeshi population of Tower Hamlets. NEON 1 follows a sequential design for evidence generation, beginning with a systematic review series of the literature on infant feeding and care practices in Bangladeshi and other SA families living in: India, Pakistan, Bangladesh and high income setting [28-32]. This paper presents the results of the subsequent qualitative formative study phase, with participants from 'community', 'key informant', 'health professional' and 'family' groups (across generations), as informed by the multiple levels of the socio-ecological framework [33]. Evidence from the literature and qualitative study informed an adapted Participatory Learning and Action (PLA) group approach [34] to optimise infant feeding and care practices amongst Bangladeshi infants aged 6 to 23 months and will be described elsewhere.

A Community-Based Participatory Research Partnership

Informed by a Community-Based and Participatory approach [35,36], Community Facilitators (CFs) were active partners in study design, data collection, analysis and interpretation, towards intervention development. Community facilitators worked as partners in the study team and as a bridge to the community, helping to facilitate engagement, crossing language barriers and informing accessible and appropriate topic guides. CF roles were advertised through local community organisations and parent networks. CF recruitment criteria were a) adult over 18 years of age, b) parent, c) self-identified as being of Bangladeshi heritage and d) living in the local area at the time of the study: Tower Hamlets, London. Two CFs (one male, one female) were selected via interview according to their awareness and motivation towards the study and topic. CFs received an orientation day, a refresher day and two days of full training on interview and facilitation skills, data confidentiality and informed consent.

Patient and Public Involvement

The NEON study was initiated in response to a clinical need identified by the UK SA community and health care professionals. A co-production prioritisation exercise conducted with said groups identified the need for further research into obesity and diet within the UK SA population [37]. Furthermore, the epidemiology of the British-Bangladeshi community in TH and importance of

For peer review only - http://bmjopen.bmj.com/site/about/guidelines.xhtml

the first 1000 days of life in relation to child growth and development provided a strong rationale for research and subsequent intervention with this population. Finally, the TH Clinical Commissioning Group (CCG) specifically identified the complementary feeding (CF) period (from 6 to 23 months) as an area lacking support and interventions within the borough, with breastfeeding and child health already addressed through a range of borough-wide services and interventions. Once the need was identified by both the community themselves and the local CCG, Patient Public Involvement (PPI) representatives were identified and engaged in every step of the study from protocol development, to study design and dissemination of the results.

In addition to working in partnership at every stage with the community facilitators, PPI representatives were invited to attend quarterly research meetings and provide cultural oversight and study accountability. PPI study representative roles were advertised through local community organisations and parent networks. PPI study representative recruitment and selection criteria were the same as the CFs. Two PPI study representatives (both female) were selected.

CFs and PPI representatives received high street vouchers to thank them for their involvement and travel costs. The value varied according to length of the activity and guidance from community-based research partnership experts and involvement.

Community and health professional dissemination workshops were conducted in June and September 2019 respectively, with study participants invited to attend. Key findings, recommendations for an adapted PLA group approach and next steps were presented and discussed with workshop participants, allowing for verification of identified themes and identification of ways to move forward to address suboptimal infant feeding and care practices and contributing factors within the study population.

For peer review only

Study Population

The study is located in the London Borough of Tower Hamlets (TH), an area with the largest British-Bangladeshi population in the UK and one of the most socio-economically deprived of any London borough, scoring the highest rates of child poverty and unemployment [38]. Sylheti is the most common language spoken by British-Bangladeshi residents of TH but is not a written language, meaning health promotion materials are usually written in English or Bengali and therefore limiting access to important information.

Participant recruitment

Multiple recruitment strategies were used to purposively sample the British-Bangladeshi population of TH. CFs shared study details with local schools, community and children's centres, and NHS clinics within different districts in TH ranging from Whitechapel to the Isle of Dogs. To ensure access to families that might not otherwise access health services, CFs also actively recruited from public spaces and through informal networks and through word-of-mouth. Posters were developed in English and Bengali, and placed in public spaces, however, CFs were encouraged to recruit via word-of-mouth to overcome language and literacy barriers. Key Informants were identified during community focus-group discussions (FGDs) and through snowball sampling. Health professionals were identified and contacted by the researchers. Potential participants were approached by CFs in person or via telephone and provided with information and expression of interest documents. CFs arranged a venue, time and date convenient to participants, utilising community centres, children's centres and other public spaces in TH. The researcher organised all financial costs related to room bookings, transport and snacks.

Sampling and inclusion

Study participants were eligible if they self-identified as British-Bangladeshi and were resident to TH, with the exception of health professionals who were only required to practice in TH. Inclusion criteria further differed by participant type. Community members were not required to have children in the study age range (6-23months). Community key informants were included on the criteria that they held a significant role in the community. Health professionals were eligible if they had experience of nutritionally-related diseases amongst infants and young children in TH; professions represented in this study included health visiting, midwifery, speech and language therapy, dentistry and GP. Finally, participants of the family phase were eligible if they were: a) pregnant or b) caring for a child aged 6-23months in the capacity of: mother, father, caregiver or grandparent. Pregnant women and mothers were stratified according to time spent living in the UK (< or > 3years).

Focus group discussions

Focus Group Discussions (FGDs) were first conducted with community members to explore social norms towards infant feeding and identify stakeholders influencing infant feeding and care practices in the target population. FGDs were later conducted with fathers and grandparents to gain a consensus of understanding and inform findings from semi-structured interviews (SSI) conducted with mothers and pregnant women. As advised by CF/PPI, all FGDs were separated

For peer review only - http://bmjopen.bmj.com/site/about/guidelines.xhtml

by gender and status, i.e. 'fathers', 'grandmothers' and 'grandfathers' to ensure a comfortable environment for discussion. FGDs were co-facilitated by the researcher Lorna Benton (LB) and a CF. The gender of the CF was selected to match that of the FGD participants. One person acted as facilitator whilst the other took observational notes and this varied according to the study phase and group. At first, the researcher (LB) facilitated the groups but as the study progressed, CFs were capable of running the FGDs and the researcher observed. The researcher led the informed consent in English and CFs translated into Bengali or Sylheti. FGDs were conducted in community centres around TH and lasted approximately 90-120mins. Audio recordings were taken for transcription and translation and all recorded interviews were then destroyed.

Semi-structured Interviews

Semi-Structured Interviews (SSIs) were conducted with key informants, mothers, pregnant women and healthcare professionals because this approach allowed a more in-depth exploration of infant feeding and care practices and influencers amongst target beneficiaries of this study. We sought to explore more in depth cultural and social norms towards infant feeding. These norms may change after migration to a new country [39] and thus we stratified recruitment of mothers and pregnant women by migration status: based in the UK <3 years or >3years. The CF typically led key informant, mother and pregnant women SSIs in English, Bengali, Sylheti or a mixture of languages in community spaces or in the person's own home. Health professional interviews were conducted in English and in a NHS space or via phone, by the researcher (LB). SSIs were conducted individually except for one mother interview, where her mother and sister also attended. SSIs lasted approximately 45-90minutes and were audio-recorded.

Topic guides

Topic guides followed a consistent structure but were tailored to the participant to encourage a progressive depth of information discussed. The literature [28-32] informed community and key informant and health professional interview tool guides. 'Family' topic guides were modified based on findings from these prior phases and tailored to family members (e.g. pregnant woman or mother). Participants were asked their thoughts on what foods they consider as healthy, and what a healthy child looks like and why, except for health professionals, who discussed influencing factors and health consequences encountered in their profession. Prompts were prepared and used for social, cultural and environmental influences on infant feeding. Guides were developed in English and translated into Bengali. Translations were checked and edited by

For peer review only - http://bmjopen.bmj.com/site/about/guidelines.xhtml

CFs to ensure reliability and consistency. CFs were prepared to speak in Sylheti, Bengali or English, as per the needs of the participants.

Film

Stories and anecdotes collected during the early phases of data collection were used to produce a short film, introducing the topic of the study and stories to parents and caregivers that might be otherwise difficult to share to e.g. the narrative from one mother of filling a bottle of milk with crushed biscuits to 'top up' her infant feeding. The film was used to help with ice breaking during individual interviews with parents and pregnant women. The use of film has been recognised as a useful tool to support participants in feeling less threatened whilst yet familiarising them with the issue to be discussed. The films were collected in partnership with the community and only those films were used where consent had been provided for use in such settings

Ethical considerations

During FGDs and interviews, the researcher led the written informed consent in English and community facilitators translated into Bengali or Sylheti. The information sheets were translated into audio recordings in Bengali and Sylheti dialects. Translation and recording of study materials were carried out by CFs. The project was approved for research ethics from the University College London Research Ethics Committee (Ethics project ID number: 10271001) and the Health Research Authority (Rec Ref 16/EM/0134).

Qualitative Data Analysis

Transcripts were checked by the researcher for fidelity of the topic guide and detail of prompts as data collection progressed. The steps of a framework analysis process were followed, as outlined by Braun and Clarke [40] and included: transcription, familiarisation with the interview, coding, developing a working analytical framework, applying the analytical framework, charting data into the framework matrix and interpreting the data. Transcriptions were completed by the CFs and outsourced to a professional company, parallel to data collection. CF and the researcher (LB) discussed and agreed when data saturation had been achieved. A combined inductive and deductive approach was taken to allow for socially located themes in the assumption that unspecified traditional and cultural beliefs are likely to influence feeding practices in this population. Two researchers independently familiarised themselves with the data and discussed preliminary themes and codes, to decide on the final themes and subthemes. Researchers applied

For peer review only - http://bmjopen.bmj.com/site/about/guidelines.xhtml

open codes to the transcripts using NVivo 11 [41] and sought CF input into thematic analysis, to revise themes where applicable.

For peer review only

RESULTS

A total of 141 participants were recruited to 12 focus groups and 45 semi-structured interviews (Table 1).

Table 1: Characteristics of study participants

Participant	FGD / SSI	Sex	N (Total)	Language
Community members	FGD (n=6)	Female	7	Sylheti
		Male	8	English with some Sylheti
		Female	5	English with some Sylheti
		Female	10	Sylheti
		Male	7	English with some Sylheti
		Female	10	English with some Sylheti
Key Informants	SSI	Mixed	6	Mixed
Health professionals	SSI	Mixed	9	English
Mothers	SSI	Female	21	Mixed
Pregnant women	SSI	Female	9	Mixed
Fathers	FGD (n=2)	Male	6	Mixed
		Male	4	Mixed
Grandmothers	FGD (n=2)	Female	14	Mixed
		Female	14	Mixed
Grandfathers	FGD (n=2)	Male	6	Mixed
		Male	5	Mixed

FDG: Focus discussion group

SSI: semi-structured interview

For peer review only - http://bmjopen.bmj.com/site/about/guidelines.xhtml

Two overarching themes were identified during interviews and group discussions: (1) modifiable infant feeding and care practices that participants suggested could be targeted in order to optimise infant feeding and nutrition; and (2) socio-ecological factors believed by participants to influence these modifiable feeding practices. The Supplementary Table illustrates the themes identified and example quotes from focus groups and interviews with participants.

Supplementary Table*: Themes identified and example quotes from focus groups and interviews with participants 'Supplementary Table'*

For peer review only

For peer review only - http://bmjopen.bmj.com/site/about/guidelines.xhtml

For peer review only

For peer review only - http://bmjopen.bmj.com/site/about/guidelines.xhtml

Theme One: Modifiable Infant Feeding and Care Practices

Figure 1 depicts the four sub-themes identified, namely: 'early and late introduction of semi and solid foods'; 'overfeeding'; 'feeding to fill the belly' and 'prolonged parent-led feeding practices'. Within some of these sub-themes, there were further connecting sub-themes as depicted in Figure 1. For example, overfeeding included four connecting sub-themes: forced feeding, unregulated portioning, top-up feeding and distraction feeding.

Figure 1: Connecting sub-themes related to Theme One: Modifiable Infant Feeding and Care Practices

Early and late introduction of semi and solid foods

Parents were highly aware of NHS recommendations to introduce semi-solid and solid foods at six months. We identified differing attitudes towards this recommendation, favouring early (3-5 months), timely (6months) and late (7-8 months) in different households, and a sentiment that specific advice regarding timing is '*always changing*'. Early introduction was favoured amongst many participants who considered that early introduction would improve the nutrition of non-breastfed babies or help their infants to grow and develop faster. Food advertising was given as a reason for the early introduction of solids from four months of age in a few households. Conversely, only a few parents introduced solid and semi-solid foods later than 6 months and did so because they perceived milk as nutritionally sufficient. Late weaning was not as typical as early weaning.

"*As a Bengali we tend to put our children onto solid food as quickly as possible*".

-Mother

For peer review only - http://bmjopen.bmj.com/site/about/guidelines.xhtml

Reasons cited by caregivers to introduce solid foods (irrespective of timing of introduction) included: the personality of the child, showing interest in food, health professional advice, when infant starts putting things in their mouth and when the infant is sitting up or crying for food.

Overfeeding

Awareness on how to regulate feeding portions was mixed, with some parents stating that infants are able to self-regulate and others stating they '*don't have the understanding of fullness*'. Four approaches to feeding are described below under the sub-theme of overfeeding.

1. Forced feeding

Key informants and health professionals defined "forced feeding" as consistently encouraging a child to eat when not hungry. Parents did not report force-feeding themselves, but some said they had seen examples in their family or community. For example, holding a child in arms and feeding via parent-led practices, when the child is not thought to be hungry. As a nuance to the theme, several mothers and fathers reported parents need to '*work hard and not force the child*' because it can be an unpleasant or stressful experience and risks making children afraid of food. Those parents said they stopped feeding when the infant/child pushes the spoon away, refuses food, says 'no' or closes their mouth.

2. Un-regulated portioning

Family and community participants agreed that portion size is not customarily measured and often based on intuition. Health professionals reported that portion size can reflect adult portioning and that parental awareness, and a high frequency of feeds are barriers to age appropriate portioning. Parents felt that children were not eating enough volume of food when they took 'a half portion' or '2-3 spoons' per feed. One health professional addressed this concern in their own clinical practice (unrelated to this study) by filming in several households and measuring portion size and comparing perceived portion size with measured portion size. They found that measured portion size differed with perceived portion size frequently underestimating actual portion size (personal communication). Exceptions were found, however, amongst some parents, usually those that had received support from health services or sought information online.

For peer review only - http://bmjopen.bmj.com/site/about/guidelines.xhtml

3. Top-up feeding

Regular 'top-up' feeding was defined as the practice of offering frequent and additional meals to an infant's feeding routine. Meal timings varied between households with a tendency to eat late into the evening (8pm or later), providing time for extra feeds before infant/child bedtime. Some parents in this study said they gave their children milk and sometimes solid (jar) food immediately before the children go to bed. Occasionally, caregivers reported adding sugar or biscuits to milk bottles to encourage calorie intake between solid feeds.

"The other thing is obviously a lot of the people say right in our community, which is true, people eat up to the brim of their neck and even sometimes you will see people eating just after 15 minutes before they go to bed which is not good because you have not digested your food yet, it is still in your chest."

- Community member

4. Distraction feeding

Distraction feeding described the caregiver's practice of distracting the infant/child with videos on the phone or TV whilst feeding directly, e.g. via hand or spoon. Parents reported mixed attitudes towards the importance of play during meal times: some felt they benefitted by avoiding messy eating and from the convenience of using distraction techniques, whilst others viewed this as a missed opportunity for interaction between caregiver and child. The TV was perceived as a helpful tool for grandparents with limited mobility to spend time with a child and supported mothers to undertake other household chores. A smaller number of caregivers described other distraction techniques including singing and storytelling whilst at the same time allowing or encouraging the infant to make a mess at meal times.

"[...] you know some babies they don't like to eat, almost you have to make different circumstances to make them happy to eat, like some entertainment thing like rhymes or something. Or lots of toys to put in front of her high chair and then [...]"

- Mother

For peer review only - http://bmjopen.bmj.com/site/about/guidelines.xhtml

Feeding to fill the belly

Community and family members described how caregivers often feel a need to *'fill the belly'*, to ensure good growth and development. Some health professionals elaborated on this theme from their experience of parents that place an emphasis on feeding 'enough'. At most extreme, this can be taken as a literal demonstration in which community members said that caregivers might look for a physical protrusion of the belly. Awareness of fussy eaters appeared to motivate some parents to practice a range of approaches during mealtimes.

"There have been times, many, many times we've been telling parents how their baby's stomach is probably this small, like the size of their fist, that's how big your stomach is. So, if you are trying to feed them that much rice on a plate, that is more than they should be taking in. Parents tend to think oh no she didn't eat properly, she hasn't had 5 bites and they want to give more bites in her mouth or encourage to eat more. But really that is all the food she can take."

- Community member

Preferences for milk and sweet foods

A desire to fill the belly influenced the types of food and drink that parents preferred to give their infants, introducing formula milk, high energy (prioritising rice-based foods) and sugary snacks (e.g. yoghurt, banana, crisps or other snacks).

"I never know, my husband always says that's enough, don't give no more. He likes you know Asians make this thing called Kheer, it is milk with rice and sugar and it is really sweet and it is really nice. He absolutely loves it. Any time of day you feed him that he'll eat it."

- Mother

Community members, informants and some parents recognised sugar as a customary part of the British-Bangladeshi diet. Community informants described a tradition to provide dates as the first food. Families described how sweets and treats are customarily shared by household visitors, and health professionals addressed the consequence that treats are frequently visible or on display in the household. Some mothers reported treats, including custard tarts and biscuits or

For peer review only - http://bmjopen.bmj.com/site/about/guidelines.xhtml

fizzy drinks, were commonly shared with young children. On the contrary negative attitudes to fizzy drinks did prevail amongst some study participants.

Mothers reported regularly preparing fruit juices, either as fresh, using a blender, or purchased, for example mango juice from concentrate. Caregivers considered homemade smoothies and fruit juices as a healthy breakfast, lunch or snack but health professionals connected these preparations to a high daily sugar intake. Health workers described a tendency to wean onto the bottle using fruit juices and fruit squashes. More informed participants were aware of the risk to oral health posed by sugary diets, but still felt unable to break their dependence on sugar.

"I just don't want him to have too much sweet, because obviously all the kids in my family had all teeth taken out. I don't want him to go through that, he's only got five. I don't want him to lose all his beautiful teeth. I try and keep him away from sweets. I say that but I want to keep him away from sweet stuff"

- Mother

Chasing fussy eaters

The phenomena of fussy eaters were described by participants at all levels as: children running away from or denying food, avoiding vegetables, avoiding solids, sticking to one kind of food or only taking three spoons against an expectation of four or five. Commonly described scenarios were of parents following children around, trying to feed them with their hands. Self-proclaimed parents of fussy eaters described their anxiety and concern for the dietary diversity of their children.

"You put him in his highchair and give him a few spoons and that's it. No, turns his face. And I have to like literally beg him, please one more, one more, he won't eat. And he's quite a healthy child. You'd think he eats quite a bit, he doesn't. He drinks 8ozs of milk and for him that's enough, he doesn't want no more."

- Mother

For peer review only - http://bmjopen.bmj.com/site/about/guidelines.xhtml

Parents shared a range of concerns around the potential future health of their child and some spoke to the risk of Vitamin D and iron deficiencies when asked if they provide their child with any supplementation. A concern around the risk of Vitamin D deficiency stimulated one mother to switch to formula milk. Respondents from the community, health professional and family groups pointed to a cycle of fussy eating and the consumption of milk rather than solid foods. Health professionals observed that meal times can last around an hour, or could be replaced by sugary snacks and treats, perpetuating a cycle of milk and sugar but no meal. They recommended early exposure to a variety of textures and repeated attempts to introduce new foods (for example, they described routinely advising families that it can take up to 14 attempts to accept a new food item) and advised against distraction feeding as a risk towards over-feeding.

"*[…] especially in the Bangladeshi community they have a Vitamin D deficiency so mum milk has everything but if the mum doesn't have enough vitamin D, the baby is not going to get enough vitamin D, so doctors are saying that formula milk has vitamin D, so mixed feeding is good. I talk to the doctor and my health visitor, they all listen to me. The formula milk has vitamin D specially made for the deficiency and so yes, I need to give him formula milk.*"

- Mother

Prolonged parent-led feeding practices

Infant feeding and care practices were predominantly parent-led (hand feeding, spoon-feeding, bottle-feeding, syringe) with few parents practicing infant-led (i.e. with finger foods) feeding.

Prolonged hand and spoon-feeding

Hand feeding was practiced by parents as a symbol of love from parent to child and because they felt it could improve the taste of food whilst also allowing parents to remove small bones and to avoid messy feeding.

"I think because my Mum's always hand feed us, even when we were quite old she would hand feed us, […] I'm sure that Mum's here have hand fed their kids […] my son is five years old and I will hand feed him because rice and curry is mostly hand fed. Also, it's the love as well, it's the love aspect. It's like you're showing them you love your child, so then you feed them more. More than the love aspect is the easiness […] instead of them making a mess you just feed them […]"

- Community member

For peer review only - http://bmjopen.bmj.com/site/about/guidelines.xhtml

Health professionals and some parents raised concerns for extensive hand and spoon-feeding over a prolonged period because it is considered a missed opportunity to encourage self-feeding practices and cognitive-motor skill development. The social impact was described by health professionals in a commonly described scenario, where some British-Bangladeshi children were described as being unable to use knives and forks when they reach primary school.

"Culturally we've always eaten rice by hand, okay. That's something I would never let go of. In all honesty, rice and curry is so tasty with hands, we don't enjoy it with fork and knife."

- Mother

Spoon-feeding was preferred for semi-solids, such as porridge or kichadi (a rice and lentil preparation) or during teething, whilst the syringe was less commonly used and mostly for water when the child was considered to have digestion problems. Parents using beakers were also following advice to introduce at 6 months and often use it for water but also used it for juices such as Ribena. Infants were fed in a range of positions, often depending on the method of feeding and location. Inside the home infants may sit in a high chair at the table or separately, on the lap, floor, sofa or caregiver's arms. A common scenario was described of '*chasing the baby around the sofa*', although this was seen as less common.

"I saw most of the mothers running behind children with a spoon"

- Mother

Prolonged bottle feeding

Bottle-feeding was frequently reported amongst British-Bangladeshi families in this study. Parents reported that the bottle was introduced as early as a few weeks or months in households that followed a mixed (breast and bottle) feeding approach. Occasionally, parents were motivated by a belief that formula milk is scientifically prepared and thus more beneficial for the child. Many mothers introduced the bottle after birth to facilitate feeding outside of the house, during social situations or when visitors come to their home. Typically, the bottle was viewed as a

For peer review only - http://bmjopen.bmj.com/site/about/guidelines.xhtml

stepping-stone towards the introduction of solid foods. Friends and family often encouraged bottle-feeding to ensure the 'belly is full', as it was felt to allow the possibility of more frequent feeds, such as an extra feed before bed to help the infant/child sleep and allows family members to help free up the mother's time.

"My boy is used to the bottle from his father, maybe at just one or two months he did breastfeeding, after that he didn't breast feed now he is sleeping my wife tried the breastfeeding at night time. Sometimes he drinks but sometimes not, but he used to have the bottle and it's easier for us as well to give him the bottle."

\- Father

There were various accounts of how community members who had seen the bottle being used in a suboptimal way for example, the contents being modified to include sugary additives, such as biscuits, or to give juice such as mango to infants less than 6 months of age. One community member recalled a friend had left a bottle of juice in the mouth of an infant overnight, something that she was concerned about for the impact that it could have on tooth decay. Another community member described 'modified bottle' practice included cutting the teat of the bottle to feed semi-solid foods.

One health professional recommended a complete transition away from the bottle by 1 year of age, but a form of prolonged bottle-feeding was described by health professionals amongst children up to the age two or three years old in many of the households. A speech and language professional identified prolonged sucking on a bottle teat as a risk factor for impeded devolvement in the roof of the mouth of some British-Bangladeshi infants in their care. Health professionals also expressed concern that prolonged dependence on the bottle may suppress the appetite and that it is linked with an additional issue of dietary diversity of children consuming excessive milk.

"A third of our children are on the SEN [special educational needs] Register which is speech and language. They are still probably having bottles, they are probably still on dummies and they probably aren't eating solid foods properly and they are being (hand) fed as well. That's the other thing in our community as well that children up until the age of about 7 or 8, even older, are physically fed rice, you know, hand fed. When children come into nursery […] some of them don't

For peer review only - http://bmjopen.bmj.com/site/about/guidelines.xhtml

even have a clue about how to use a knife and fork because they have been fed. When the food is fed […] rice is mushed with their hand […] it doesn't give them that whole experience of the chewing."

- Health professional

For peer review only - http://bmjopen.bmj.com/site/about/guidelines.xhtml

Theme two: Factors influencing infant feeding and care practices in the British-Bangladeshi population

Figure 2 illustrates the wider socio-ecological factors identified by participants that they thought impacted on infant nutrition that fell into three subthemes as depicted in Figure 2: (1) society and culture, (2) physical environment and (3) information and awareness.

Figure 2: Connecting sub-themes informing theme two: Factors influencing infant feeding and care practices in the British-Bangladeshi population

Influences from society and culture

Chubby equals healthy

Community members, key informants and parents presented an archetypal societal image of a 'chubby', healthy child. Community members rooted this finding in a belief that extra weight safeguards growth and development during childhood – and this is something that was echoed in the discussions with parents. Parents placed extensive emphasis on good growth and development, which was experienced by health professionals as a concern for appropriate growth along WHO growth centiles. Health visitors described having guided some mothers to develop a routine to support their meal planning and build resilience against outside community perceptions of a healthy weight. Some parents have learned about the importance of responsive feeding by attending local courses. Community members explained that past generations had experienced a scarcity of resources when raising children in rural Bangladesh and this legacy continues to influence modern feeding practices, although resources are greater and physical activity is lesser in urban East London.

"The baby who has good health with no disease, is chubby. With good health they look nice."

\- Pregnant woman

For peer review only - http://bmjopen.bmj.com/site/about/guidelines.xhtml

Anxiety and social visits

Mothers reported some anxiety towards social occasions and particularly if they anticipated comments from extended family members or friends, suggesting their child was too skinny, as a critique of not feeding enough, or too chubby, when perceiving jealousy [see quote]. Mothers reported occasionally finding it difficult to challenge social practices, such as offering treats in other people's households, even when they avoided such norms in their own homes.

"If we try to talk to her about [overfeeding her child] she gets offended. In Bengali culture, we have what we call the 'evil eye' [...] so, she thinks we are giving her children the 'evil eye' because they eat well, they eat a lot. We still talk to her about it [overfeeding] though, we don't stop."

- Mother

Physical and local environment

Physical space for play

Community members, especially fathers and grandfathers, felt that limited household space left little opportunity for play in some households. The limited physical activity of most family members was often considered to begin in childhood.

"If you want to be healthy you've got to start from the beginning. Here what do you do? People are living in a box, just in the house or flat, got very limited space for a young child, 9 months or 1-year old child, it's time for them to crawl around. Is there any space to do that? No. Their bone structure, everything, is not getting stronger because they are not exercising, they are not moving around that much. What they do, say put them in a pushchair, in front of the TV and that's it."

- Community member

Elevated levels of outdoor air pollution and concerns for safety in public spaces on rare occasions were added barriers to exercise. Furthermore, exercise was not considered part of the daily routine, this being enhanced by a recognition of the shift in lifestyles after migration from very physical in rural Bangladesh to more sedentary lifestyle in urban UK. However, some

For peer review only - http://bmjopen.bmj.com/site/about/guidelines.xhtml

parents did encourage swimming, football or taking walks in the park, facilitated by access to facilities and female-friendly sessions to try and encourage some engagement with exercise.

Fast food outlets

Community members widely regarded the abundance of fast food outlets in the TH environment as a well-established influence of takeaway meals such as chicken and chips. Some parents reported having a weekly takeaway as a treat. Families also tended to share foods with infants/children and allow them a small taste of these meals which were often high in salt and fat.

"[…] my grandchild is nearly 6 years old and we did try a little bit of chips to taste it."

- Grandfather

Advertising

Parents found the advertising of semi-solid and solid convenience foods from the age of 4 months as confusing and indicative that it is an appropriate age to try solid foods. The availability of formula marketed for 'hungrier babies' supported some parent perceptions that their baby needed more food or more energy dense foods than other babies.

"[…] I think anyway if you go to the supermarket and you see something that says four months then you should be doing that because the packaging, if the baby food companies are telling you that you should be feeding your baby at four months why wouldn't you, why would you wait until they were six months, because even though it is not that much of a difference but when you have had a baby they are growing so quickly you think that two months is a big deal and that you should be giving that extra food, but definitely I do not think many families that I have seen will wait until six months."

- Community member

Household environment

The visibility of fruit and vegetables in the household was seen as an easy barrier to overcome in many households. Health professionals and key informants within the community observed that fresh fruit and vegetables are not usually on display in households. Conversely, sweets may be

For peer review only - http://bmjopen.bmj.com/site/about/guidelines.xhtml

on display in the home during social situations such as family gatherings. Health professionals advised helping to introduce different food types and textures by allowing for the visual and touch experience, beyond just taste.

"[…] Especially around holiday time everyone goes to everyone's houses and things like that. A lot of the dishes would be made, obviously when they are doing these kinds of things you are not looking at what you are cooking, what kind of ingredient is going in, what level of salt or sugar has gone in. That is when children do eat quite a lot more than what they are supposed to eat when you have got guests coming into your house they are bringing like sweets, crisps and things like that for your children."

- Key informant

Information and awareness

Engaging with health services

Mothers and Fathers differed in health seeking preferences; fathers stated a preference to speak to a GP whilst mothers described preferring to seek information from sources including Health Visitors, Children's centres, online and community groups. Health professionals and informed community members reported a range of demonstrations and activities for new parents offered at local health and community centres and fundamentally supported with a crèche facility. However, parents and carers widely perceive that advice is constantly changing or conflicting or simply not available. Pregnant women felt antenatal classes would help to explain what to do but not how to feed after birth and fathers sometimes felt ignored by GP's. In general, parents reported wanting more support on topics ranging from: how to introduce solid foods, when to introduce semi/solid foods, when to stop giving milk, what foods to give, in what ratio and how much.

"I am always confused, I am always ringing up the doctors and asking them questions all the time, especially about the milk thing. They said it depends on your child, every child is different. I was a bit like he's still little, doesn't he need the nutrients in the powder to grow?"

- Mother

First time parents especially wished to have more information and support. Health professionals reported that complementary feeding and care needs to become a routine aspect of post-natal

For peer review only - http://bmjopen.bmj.com/site/about/guidelines.xhtml

care services and that many would benefit from a more consistent '*hand-holding'* approach that is currently lacking.

Balancing cultures

Mothers often tried to interpret information from different cultures and generations in their infant feeding practices. Grandparents and in-laws were viewed as either supportive influences or pressurising depending on the household. Parents reported adverse feeding practices amongst extended family members, including: feeding extra meals, feeding before bed and placing children in front of the TV.

"By starving, I actually used that term, starving may mean they think the child has not eaten [...] like for example if they have not eaten in two or three hours [...] I will give you a perfect example, I went to the wedding over the weekend and my mother in law was on my case to feed my son [...] I just said when he is hungry he can communicate with me, he will come up to me and say mum I want to eat. I tried three times, after three times I am not going to go round walking after him. My mother in law you know how she is she is so protective of her grandchildren […] she took him and she fed him, so can you see the difference?"

- Mother

Grandparents reported having usually experienced very different advice and support whilst they were parenting their own children, to parents living in the UK and this appeared to create a generational gap in knowledge, lived experience, awareness and practice that was recognised by both generations. Some grandparents reported feeling like their children did not listen to their advice whilst some mothers and carers reported their mothers or mothers in law provided conflicting advice to that of health professionals. However, these opinions were not universal.

"I remember before I got married before I had my kids they used to do what my mum did which my mum did twenty three years ago, twenty four years ago, at that time it was just egg custard and stuff so they did not know much about vegetables, they did now know much about a lot of stuff. Even when we were growing up probably my mum gave like at six months they started introducing what they ate but before that they gave more like egg custards"

- Mother

For peer review only - http://bmjopen.bmj.com/site/about/guidelines.xhtml

Some mothers felt they needed to be '*very resilient'* to continue breastfeeding, whilst others felt that grandmothers or mothers in law were supportive of exclusive breastfeeding. Commonly, however, many mothers were proactive in seeking information from different sources, and some mothers felt they were able to resist unhelpful advice by '*being a strong person'* and '*doing my own research and asking advice'*, or by having a close network of experienced mums.

"[If] they are fussy eater, it is just because they were fed on only milk because of my daughter's in laws, they are afraid that house will be a mess. They do not know the foods, you have to try foods to know."

- Grandmother

Parent style

Mothers described experiencing anxiety when they felt they did not have enough or the right information and when they felt they did not know how to feed their child, a commonly expressed concern in mothers of 'fussy eaters'.

"Yes, I am worried about him because he's not eating. I try and give him, if he eats anything like the other day I gave him a choc chip roll when I shouldn't have, like it's not good for him to have and he ate it and I was so happy. He never says no to chocolate though, that's a bad thing. Milky bar, his grandparents, always milky bars, he loves it."

- Mother

A more relaxed or confident approach was found amongst mothers that considered themselves as proactive in seeking information.

"I notice in our community we have a real thing about feeding them and making sure the fact they are having a full [...] do you see what I mean but I just tend to find that be a bit relaxed, see if they take to it, if they eat or whatever. If they don't eat, come back to it, come back to the same meal, come back to that thing. It might be you try something else, they might like it but don't become stressed by it."

- Mother

For peer review only - http://bmjopen.bmj.com/site/about/guidelines.xhtml

These mothers recognised the various sources of information and invested time in engaging with different groups, either online or in the community depending on their time, locality and interest. Some mothers considered themselves as a role model to their children and tried to adopt healthy practices to that effect or developed a daily routine with the support of health visitors.

Table 2: Themes identified and example quotes from focus groups and interviews with participants 'supplementary file'

For peer review only

For peer review only - http://bmjopen.bmj.com/site/about/guidelines.xhtml

Figure 3: A framework for priority infant feeding and care practices in British-Bangladeshi communities and the contributing factors that need to be addressed under a socio-ecological approach.

For peer review only

For peer review only - http://bmjopen.bmj.com/site/about/guidelines.xhtml

DISCUSSION

Two overarching drivers of infant feeding and care practices were identified in this qualitative study focusing on the British-Bangladeshi population of Tower Hamlets, namely: potentially modifiable parent infant feeding and care practices and wider socio-ecological factors. An assortment of potentially modifiable feeding and care practices were identified that may act to restrict dietary diversity, override infant satiety, facilitate a reliance on sugary or sweetened foods and drink, and encourage over-feeding. Common parent-led feeding practices make it difficult for many parents to pick up on satiety reflexes in the infant/child. Parents often feed when they perceive the child as hungry or to show affection in common practices such as: holding a baby in arms; distracting the child with a phone/TV; and prolonged parent-led feeding (via spoon, bottle or hand); however, such practices can override childhood satiety cues and impact appetite, oral hygiene and speech and language development in childhood and across the life-course.

Figure 3 provides a thematic representation of factors that contribute to suboptimal infant feeding and care practices spanning the socio-ecological environment. Of note, the local food environment is a complex system known to shape individual diet [42]. Participants of this study described social, built and natural influencing factors of infant feeding (Figure 3). Societal perceptions of 'chubby' as healthy was an especially prominent theme and discussed in tandem with anxiety amongst those that felt they were not following normative approaches to 'filling the belly'. Participants also emphasised the benefits of access to children centres and health visitors in Tower Hamlets for accessible support and problem solving. Challenges extended to the local environment, where participants identified a lack of opportunities for play and physical activity, appropriate feeding spaces, an abundance of fast food outlets and misleading advertisements of infant foods.

Acculturation represents an important construct that explains the determinants of health inequalities in ethnic minority groups. In this study, acculturation was found to have an effect on the nutritional feeding practices in the British-Bangladeshi community. This can be seen in the generation gap between mothers and grandparents: for example, the influence from the extended family on the way the mother feed her infant and the fact that past generations had experienced a scarcity of resources when raising children in rural Bangladesh, which continued to influence modern feeding practices, although resources are greater and physical activity is less in urban East London. This generation gap is part of the acculturation of the migrant with the host country

For peer review only - http://bmjopen.bmj.com/site/about/guidelines.xhtml

where second generation were found to be more susceptible to follow the NHS and health advice. With that in mind, culturally-sensitive health information could help reduce the negative effect of acculturation and health disparities on infant nutrition and complimentary feeding practices.

Consistency

Our findings are consistent with evidence of an association between ethnicity, infant feeding and care practices and BMI amongst the British and SA populations in Bradford [43]. We add to emerging evidence from Tower Hamlets that identifies a myriad of factors, including family and friends as influencers of infant feeding and care practices in TH [44] and a systematic review that recognises the value of heritage, tradition and culture on infant feeding and care practices within SA families living in high income settings [31].

Strengths and limitations

Culturally-acceptable interventions to promote optimal infant feeding and care amongst minority populations have been relatively under-researched to date. Existing literature does not adequately capture the cultural and social determinants of infant feeding and care. A strength of this study is the development of culturally-sensitive interview tools and recruitment strategies, based on evidence from the literature and guided by our PPI and community facilitators. This strong partnership with members of the British-Bangladeshi population of TH enabled further reach and diversity of representation in this study. A limitation of this qualitative study is that a quantitative measure of dietary intake was out of scope. However, cohort studies explore the association between infant dietary intake and ethnicity [45]. Another limitation is that all data are self-reported and have the potential for respondent bias. We believe we overcame this limitation through triangulation of respondent data, a phased approach that encouraged further probing into emerging topics between stakeholder groups and by collecting data from a relatively large sample.

Implications for clinicians and policymakers

These qualitative data describe a layer of influences and add strength to the debate around regulation and marketing of follow on infant formula and foods and availability of fast food outlets. In 2016, the UK launched 'Childhood obesity: a plan for action (2016)', in which the Department of Health set the target to reduce England's rate of childhood obesity within the next 10 years. The action plan focuses on partnership with the private sector but has been criticised for an inadequate stance on regulatory measures [46]. The sugar tax was a celebrated feature of

For peer review only - http://bmjopen.bmj.com/site/about/guidelines.xhtml

the plan, but comparatively little political action has been taken towards early childhood nutrition or enforcing advertising regulations. Updated SACN guidelines emphasise educating parents and caregivers on behavioural practices relating to infant feeding, including introducing new foods, textures and diversity, yet this will take time to reach households. As such, the current model of early feeding support can be perceived as absent, untimely or confusing for caregivers and is not tailored and does not take account for cultural practices. Recommendations can therefore sometimes seen unrealistic or at odds with community practices and may have a negative impact on health seeing behavior or engagement by minority ethnic groups.

More regular contact between pregnancy and 24-months, signposting to existing and local resources and additional support in the form of facilitator led PLA evidence informed women's groups could improve the current model of infant feeding support. The economic incentive alone for early nutritional intervention in the UK is profound; treating obesity-related disease is estimated to cost the National Health Service (NHS) £6.1 billion pounds per year and the wider costs to society estimated at around three times this [47]. A growing interest in reverse innovation [48] –the concept of transferring a product or service developed in a resource poor setting to an industrialised setting [49] - has led to demonstrable cost savings for the NHS in areas from technology [50] to community mobilisation [51]. There remains a question of who and how to deliver effective community-based programmes under the NHS? Local leadership is essential; this study was fostered in part by a supportive local agenda, where the Health and Well-Being Board enshrine 'Children's Weight and Nutrition' as one of five top priorities. Similar boards provide a comparable opportunity for action and partnership with civil society and private sector if sustained by an ideology rooted in the social determinants of health.

Generalisability

This study provides an in-depth understanding of cultural and social influencers of infant feeding and care within the largest national British-Bangladeshi population and supports observations from other SA populations in the UK [43,45]. As such, these findings may be generalisable or will be able to be used to inform similar discussion with British-Bangladesh residents across the UK as well as SA populations and other ethnic minority groups. A broad range of stakeholders, including health professionals, informed our understanding of the shared environmental challenges to infant feeding and care and need for improved Infant and Young Child Feeding services (IYCF) that exist nationally. We recommend that this tailored approach to exploring IYCF practices be extended to other ethnic minority populations in the UK. Finally, while this

For peer review only - http://bmjopen.bmj.com/site/about/guidelines.xhtml

study focuses on an ethnic subgroup, the dual burden of malnutrition is a global concern. Data on IYCF indicators are limited but the data that are available show concerns for complementary feeding practises across high, middle and low-income settings that are in line with our study findings. We suggest that while many themes may be generalizable, culturally tailored approaches will be required to explore IYCF in other settings. We seek to use this formative research to inform the adaptation of a participatory and community-based intervention in an NHS context, which could have far -reaching implications for programme design and vulnerable populations in high-income settings globally.

Future research

This study identifies the need for more evidence to inform services that effectively optimise feeding and care in the early years and target vulnerable populations in the UK. We recommend for more research to support the development of evidence-based interventions that optimise feeding practices across all ethnic majority and minority populations in the UK under a community based participatory approach. We encourage culturally sensitive and community-based programming that facilitates families to discuss and challenge societal norms underpinning infant feeding practices, considerate of the whole family unit, including fathers and grandparents. More research is needed to inform an integrated participatory approach so that caregivers and community can feasibly be included in an NHS-community partnership to co-develop infant and young child feeding services. As the PLA group approach has been demonstrated to bring statistically significant improvements in maternal and infant survival through application with participatory women's groups in low and middle-income settings [26]; NEON, therefore, ultimately seeks to determine whether an adapted PLA group approach has the potential for early and effective intervention, using infant feeding as an exemplar.

For peer review only

Conclusion

This body of work generates transferable lessons to inform similar studies for adapted or tailored programmes with other vulnerable populations. It also highlights the importance of wider determinants of health and behaviours and the role for policy and environment in addressing health outcomes in general, but in particular those high risk or more vulnerable populations. We recommend that effective, culturally-sensitive support be provided to parents, caregivers and extended family members at different 'ages and stages' through evidence based and tailored infant feeding programmes. In addition, UK infant feeding environment requires better

For peer review only - http://bmjopen.bmj.com/site/about/guidelines.xhtml

regulation of marketing of foods for infants and young children if it is to optimise nutrition in the early years.

For peer review only - http://bmjopen.bmj.com/site/about/guidelines.xhtml

Acknowledgments

The authors would like to thank the National Institute of Health Research, Collaboration for Leadership in Applied Health Research and Care North Thames for funding the NEON study. We would like to thank all NEON study team members for their contribution and guidance to research activities throughout the three phases of the NEON study. All study team members made considerable contributions to research activities and decision making throughout the duration of the study and supported identification of key study findings and recommendations stated in this report. We would like to thank the NEON study implementation partners, who supported and participated in research activities and enabled the research process to proceed effectively. NEON study implementation partners included Tower Hamlets Borough Local Authority, Barts Health NHS Trust, Newham Local Authority, Women and Children First, Women's Health and Family Services, and The Breastfeeding Network in Tower Hamlets. Thanks go to the NEON advisory board, made up of researchers, practitioners, study partners and patient public involvement members who attended NEON advisory meetings, offered guidance and supported decision making on study activities. We would like to thank the NEON study's community and Participatory Learning and Action group facilitators who performed integral roles in data collection and NEON intervention delivery activities. Finally, we would also like to thank the British-Bangladeshi population in Tower Hamlets for participating in and supporting the research.

Competing interest statement:

All authors have declared that no support from any organisations for the submitted work; no financial relationships with any organisations that might have an interest in the submitted work in the previous three years, no other relationships or activities that could appear to have influenced the submitted work.

Author contributions:

- Monica Lakhanpaul (ML) was the lead investigator, conceived the idea, contributed to the tool development and drafting of the first draft of paper and edited further drafts.

For peer review only - http://bmjopen.bmj.com/site/about/guidelines.xhtml

- Lorna Benton (LB) was lead research fellow, drafted interview tools, collected the data and wrote the first draft of the publication

ML and LB are both joint first author and have contributed equally to this paper.

- Oliver Lloyd-Houldey (OLH) was involved in interpretation of data, manuscript write up & dissemination of study findings.
- Logan Manikam (LM) was involved in study conception, manuscript write up & dissemination of study findings to partners & the general public.
- Diana Margot Rosenthal (DMR) was involved in interpretation of the results & manuscript write up
- Shereen Allaham (SA) was involved in interpretation of the results, manuscript write up & dissemination of study findings
- Michelle Heys (MH) was involved in study conception, interpretation of data, manuscript write up & dissemination of study findings.

Data availability statement: The data that support the findings of this study are available from the corresponding author, Prof Monica Lakhanpaul, upon reasonable request.

Ethical approval: The project was approved for research ethics from the University College London Research Ethics Committee (Ethics project ID number: 10271001) and the Health Research Authority (Rec Ref 16/EM/0134)

Funding: This work was supported by National Institute of Health Research, CLAHRC / Barts Health

For peer review only

References

1. Schenker S: Briefing paper: Undernutrition in the UK. British Nutrition Foundation Nutrition Bulletin 2003, 28:87-120.
2. Butland, Bryony, et al. "Foresight. Tackling obesities: future choices. Project report." *Foresight. Tackling obesities: future choices. Project report.* (2007).
3. NHS Digital National Child Measurement Programme. (Jan 14, 2019). https://digital.nhs.uk/areas-of-interest/public-health/national-child-measurement-programme Date: 2006
4. WHO: http://www.euro.who.int/en/health-topics/noncommunicable-diseases/obesity/data-and-statistics).
5. Bammann, Karin, et al. "Early life course risk factors for childhood obesity: the IDEFICS case-control study." *PloS one*9.2 (2014): e86914.
6. Roseboom, Tessa J., et al. "Coronary heart disease after prenatal exposure to the Dutch famine, 1944–45." *Heart* 84.6 (2000): 595-598.Roseboom T, de Rooij S, Painter R. Early Hum Dev 2006;82:485-491.
7. Eriksson, Johan G. "Epidemiology, genes and the environment: lessons learned from the Helsinki Birth Cohort Study." *Journal of internal medicine* 261.5 (2007): 418-425.
8. Prescott, Susan, and Richard Saffery. "The role of epigenetic dysregulation in the epidemic of allergic disease." *Clinical epigenetics* 2.2 (2011): 223.
9. Langley-Evans, S. C. "Nutrition in early life and the programming of adult disease: a review." *Journal of Human Nutrition and Dietetics* 28 (2015): 1-14.
10. Lawlor, Debbie A., et al. "Associations of birth size and duration of breast feeding with cardiorespiratory fitness in childhood: findings from the Avon Longitudinal Study of Parents and Children (ALSPAC)." *European journal of epidemiology* 23.6 (2008): 411-422.
11. Gaillard, Romy, et al. "Childhood cardiometabolic outcomes of maternal obesity during pregnancy: the Generation R Study." *Hypertension* 63.4 (2014): 683-691.
12. Rechel, Bernd, et al. "The role of public health organizations in addressing public health problems in Europe." (2018).
13. Magnusson, Maria, et al. "Social inequalities in obesity persist in the Nordic region despite its relative affluence and equity." Current obesity reports 3.1 (2014): 1-15.
14. Delavari M, Sønderlund AL, Swinburn B, Mellor D, Renzaho A. Acculturation and obesity among migrant populations in high income countries–a systematic review. BMC public health. 2013 Dec 1;13(1):458.
15. Mughal Z: Resurgence of vitamin D deficiency rickets in UK. Endocrine Abstracts 2012, 28 S7.4.
16. Lawson MS, Thomas M, Hardiman A: Iron status of Asian children aged 2 years living in England. Arch Dis Child 1998, 78(5):420-426.
17. National Obesity Observatory, 2013. Public Health England. National Child Measurement Programme (NCMP): Changes in children's BMI between 2006/07 and 2011/12, p 18. In.
18. Marcenes, W., et al. "Ethnic disparities in the oral health of three-to four-year-old children in East London." *British dental journal* 215.2 (2013): E4.
19. Clark H, Coll-Seck AM, Banerjee A, Peterson S, Dalglish SL, Ameratunga S, Balabanova D, Bhan MK, Bhutta ZA, Borrazzo J, Claeson M. A future for the world's children? A WHO–UNICEF–Lancet Commission. The Lancet. 2020 Feb 22;395(10224):605-58.
20. Perez-Escamilla, Rafael, et al. "Nutrition disparities and the global burden of malnutrition." *BMJ* 361 (2018): k2252. (https://www.bmj.com/content/361/bmj.k2252)

For peer review only - http://bmjopen.bmj.com/site/about/guidelines.xhtml

21. Feeding in the First Year of Life. SACN Report. 2018 (https://assets.publishing.service.gov.uk/government/uploads/system/uploads/attachment_data/file/725530/SACN_report_on_Feeding_in_the_First_Year_of_Life.pdf)
22. Daniels, Lynne A., et al. "The NOURISH randomised control trial: positive feeding practices and food preferences in early childhood-a primary prevention program for childhood obesity." BMC public health 9.1 (2009): 387.
23. Florin, Dominique, and Jennifer Dixon. "Public involvement in health care." BMJ: British Medical Journal 328.7432 (2004): 159.
24. Department of Health. "Liberating the NHS: No decision about me, without me." (2012).
25. Crisp, Nigel. "Mutual learning and reverse innovation–where next?." *Globalization and health* 10.1 (2014): 14.
26. Seward, Nadine, et al. "Effects of women's groups practising participatory learning and action on preventive and care-seeking behaviours to reduce neonatal mortality: A meta-analysis of cluster-randomised trials." *PLoS medicine* 14.12 (2017): e1002467.
27. Nurture Early for Optimal Nutrition (NEON) study: Aceso Global Health Consultants Ltd 2019 [Available from: https://www.acesoghc.com/neon.
28. Sikorski, Catherine, et al. "Could Postnatal Women's Groups Be Used to Improve Outcomes for Mothers and Children in High-Income Countries? A Systematic Review." *Maternal and child health journal* (2018): 1-15.
29. A systematic review of complementary feeding practices in South Asian infants and young children: the Bangladesh perspective L Manikam, A Robinson, JY Kuah, HJ Vaidya, EC Alexander, GW Miller, BMC Nutrition 3 (1), 56
30. Systematic review of infant and young child complementary feeding practices in South Asian families: the India perspective L Manikam, A Prasad, A Dharmaratnam, C Moen, A Robinson, A Light, Public health nutrition 21 (4), 637-654
31. Manikam L, Lingam R, Lever I, Alexander EC, Amadi C, Milner Y, Shafi T, Stephenson L, Ahmed S, Lakhanpaul M. Complementary Feeding Practices for South Asian Young Children Living in High-Income Countries: A Systematic Review. 2018 November; 10(11)
32. Manikam L, Prasad A, Dharmaratnam A, Moen C, Robinson A, Light A, Ahmed S, Lingam R and Lakhanpaul M. Systematic review of infant and young child complementary feeding practices in South Asian families: the Pakistan perspective. Public Health Nutrition. 2018 March; 21(4):655-668.
33. Gregson J, Foerster SB, Orr R, Jones L, Benedict J, Clarke B, Hersey J, Lewis J, Zotz K. System, environmental, and policy changes: using the social-ecological model as a framework for evaluating nutrition education and social marketing programs with low-income audiences. Journal of nutrition education. 2001 Sep 1;33:S4-15.
34. Prost A, Colbourn T, Seward N, Azad K, Coomarasamy A, Copas A, et al. Women's groups practising participatory learning and action to improve maternal and newborn health in low-resource settings: a systematic review and meta-analysis. The Lancet. 2013;381(9879):1736-46.
35. Lakhanpaul M, Bird D, Culley L, et al. The use of a collaborative structured methodology for the development of a multifaceted intervention programme for the management of asthma (the MIA project), tailored to the needs of children and families of South Asian origin: a community-based, participatory study. Southampton (UK): NIHR Journals Library; 2014 Sep. (Health Services and Delivery Research, No. 2.28.) Available from: http://www.ncbi.nlm.nih.gov/books/NBK260076/.
36. Minkler, Meredith. "Community-based research partnerships: challenges and opportunities." *Journal of Urban Health* 82.2 (2005): ii3-ii12.

For peer review only - http://bmjopen.bmj.com/site/about/guidelines.xhtml

37. Manikam L, Shah R, Reed K, Santini G, Lakhanpaul M. Using a co-production prioritization exercise involving South Asian children, young people and their families to identify health priorities requiring further research and public awareness. Health Expectations. 2017 Oct;20(5):852-61.
38. London's Poverty Profile 2017, Trust for London and New Policy Institute https://www.trustforlondon.org.uk/publications/londons-poverty-profile-2017/
39. Choudhry, Kubra, and Louise M. Wallace. "'Breast is not always best': South Asian women's experiences of infant feeding in the UK within an acculturation framework." Maternal & child nutrition 8.1 (2012): 72-87.
40. Clarke V, Braun V. Teaching thematic analysis: Overcoming challenges and developing strategies for effective learning. The psychologist. 2013 Jan 1;26(2).
41. Edlund, B., and A. McDougall. "NVivo 11 Essentials. Your guide to the leading qualitative Analysis Software." (2016).
42. Engler-Stringer, Rachel, et al. "The community and consumer food environment and children's diet: a systematic review." *BMC public health* 14.1 (2014): 522.
43. Santorelli, Gillian, et al. "Ethnic differences in infant feeding practices and their relationship with BMI at 3 years of age–results from the Born in Bradford birth cohort study." British Journal of Nutrition 111.10 (2014): 1891-1897.
44. Rayment, Juliet, et al. "Bangladeshi women's experiences of infant feeding in the London Borough of Tower Hamlets." Maternal & child nutrition 12.3 (2016): 484-499.
45. Sahota, Pinki, et al. "Ethnic differences in dietary intake at age 12 and 18 months: the Born in Bradford 1000 Study." Public health nutrition 19.1 (2016): 114-122.
46. Country report for England, see Online Appendix.
47. Public Health England, 2017: Public Health England (2018). Public Health Profiles [website]. London: Public Health England (https://fingertips.phe.org.uk/, accessed 8 June 2018).
48. Crisp, Nigel. "Co-development, innovation and mutual learning–or how we need to turn the world upside down." *Healthcare*. Vol. 3. No. 4. Elsevier, 2015.
49. Bhatti, Y. A. "Reverse innovation: Create far from home, win everywhere." *South Asian Journal of Global Business Research* 3.1 (2014): 102-104.
50. Prime, Matthew, et al. "From Malawi to Middlesex: the case of the Arbutus Drill Cover System as an example of the cost-saving potential of frugal innovations for the UK NHS." *BMJ Innovations* 4.2 (2018): 103-110.
51. Johnson, Christopher David, et al. "Learning from the Brazilian community health worker model in North Wales." *Globalization and health* 9.1 (2013): 25.

For peer review only - http://bmjopen.bmj.com/site/about/guidelines.xhtml

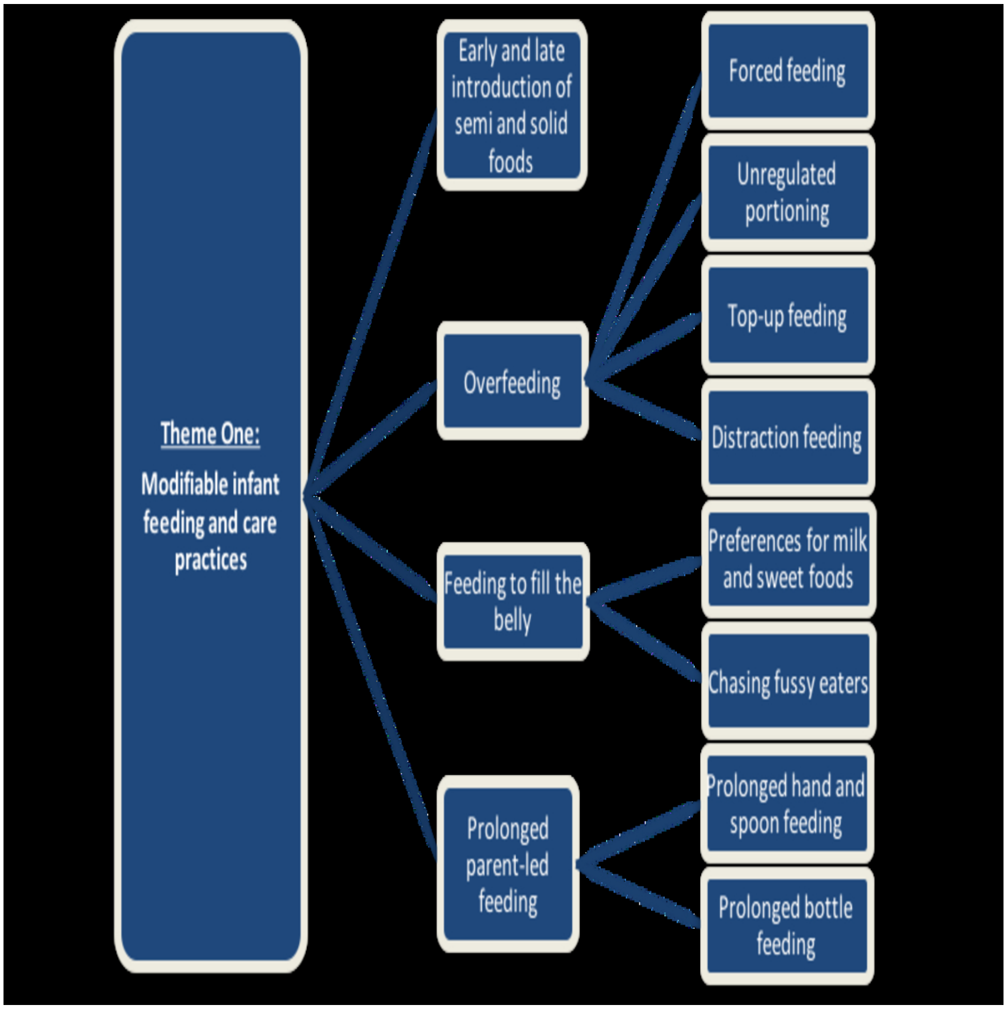

Connecting sub-themes related to Theme One: Modifiable Infant Feeding and Care Practices

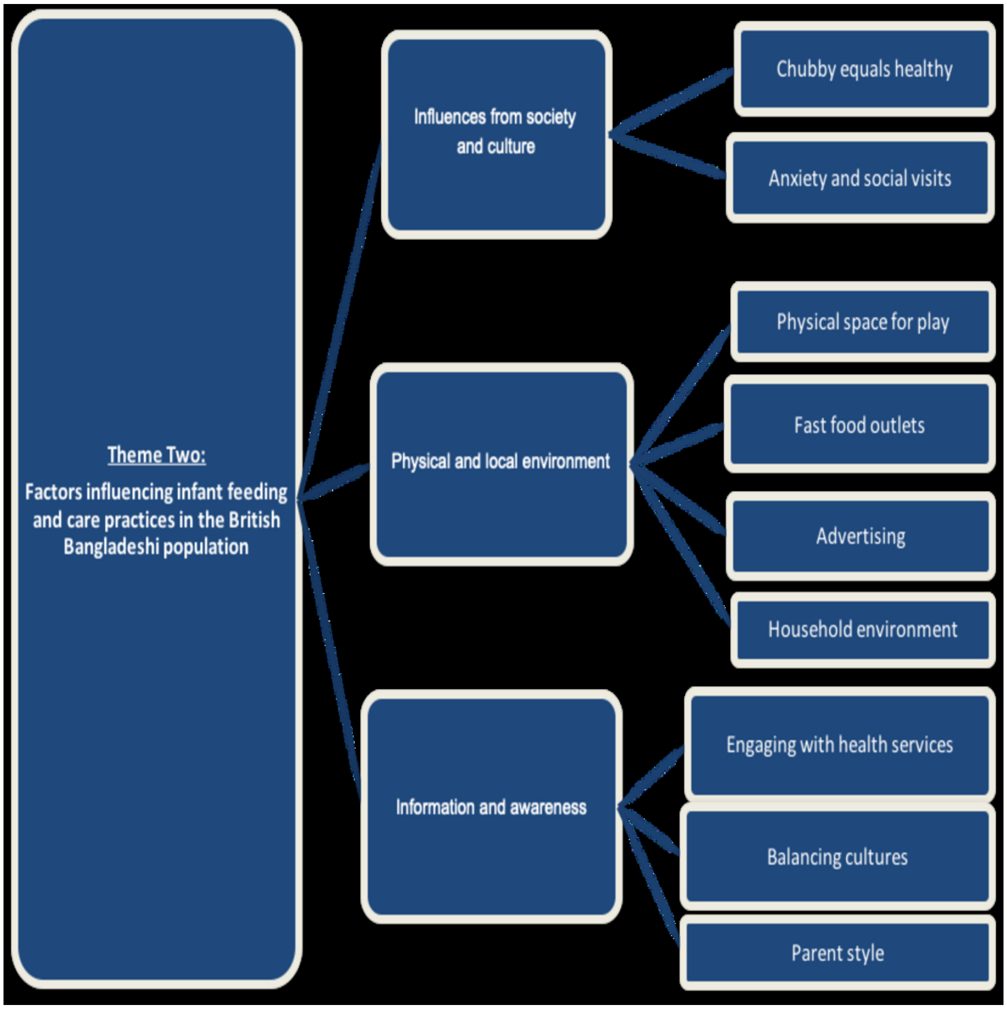

Connecting sub-themes informing theme two: Factors influencing infant feeding and care practices in the British-Bangladeshi population

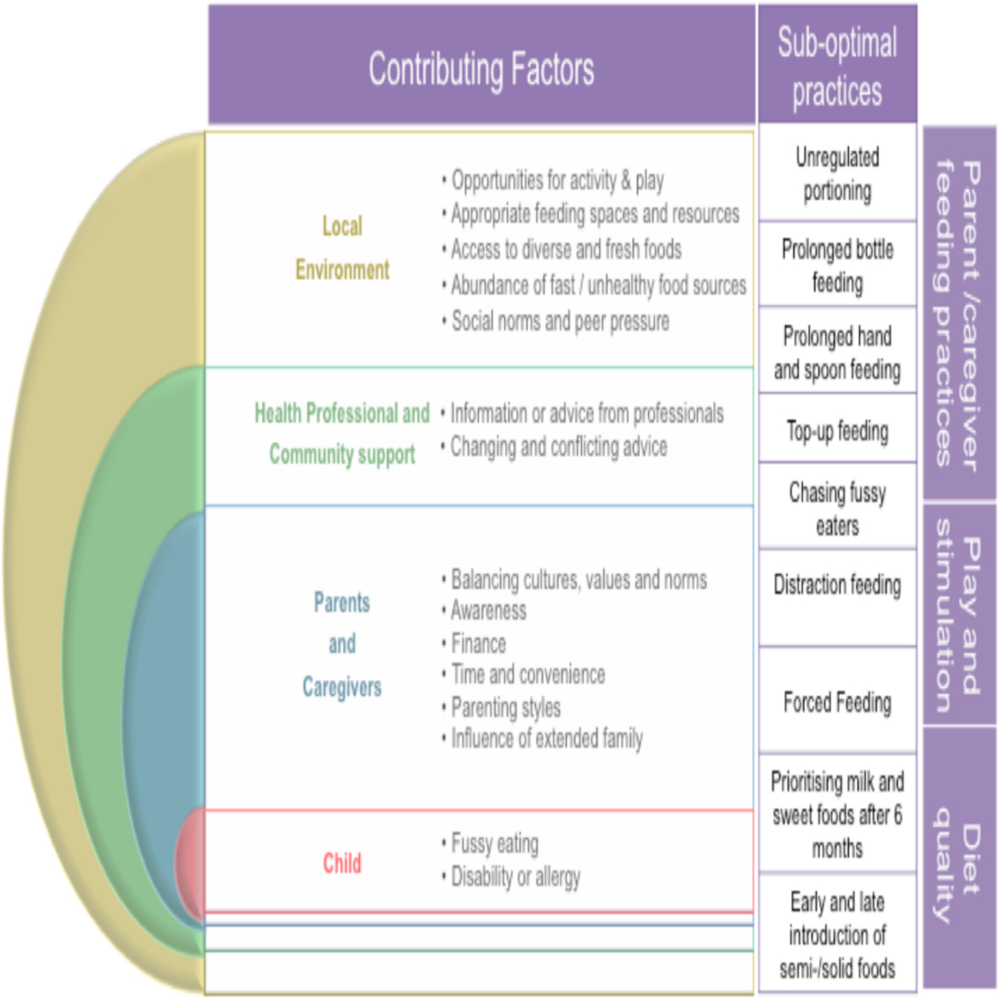

A framework for priority infant feeding and care practices in British-Bangladeshi communities and the contributing factors that need to be addressed under a socio-ecological approach.

Main theme	Sub-theme	Participants type	Example quotes
Theme One: Modifiable Infant Feeding and Care Practices	Early and late introduction of semi and solid foods	Mother	*"As a Bengali we tend to put our children onto solid food as quickly as possible".*
		Community member	*"[…] It's a cultural thing, that's what we lived on everyone, so everyone's parents say oh let's get them onto rice as quickly as possible. So that's the culture."*
		Grandfather	*"[…] most of the kids in Bangladesh they start solid food from 6 months old baby. They mash it and they give soft one. I believe in London as well they start at 6 months old and make it softer, make it mashed so children can easily eat. From 6 months they start a lot of movement and then it works to make them stronger."*
		Community member	*"What he is saying is, say for example right what you said is very, very true, what they try to do is some parents what they try to do, they give them for example breastfeeding then, 15 or 20 minutes later, the time has gone to go to sleep or something like that. Give them another bottle of milk.so obviously what*

		he's saying is overfeeding can be leading towards these kinds of problems in health issues because we just fed someone half an hour ago, why are we feeding the child again"
Overfeeding	Community member	*"The other thing is obviously a lot of the people say right in our community, which is true, people eat up to the brim of their neck and even sometimes you will see people eating just after 15 minutes before they go to bed which is not good because you have not digested your food yet, it is still in your chest."*
	Mother	*"The TV, yes he likes watching his teletubbies and stuff like that. If that is on then he sometimes does, rarely he eats, TV is his everything. If I am watching he comes up and take the remote from me. Smart like that, yes."*
	Mother	*"[…] you know some babies they don't like to eat, almost you have to make different circumstances to make them happy to eat, like some entertainment thing like rhymes or something. Or lots of toys to put in front of her high chair and then […]"*
	Health professional	*"Particularly we see lots of toddlers who are having excessive milk and not having very much solids because there's no real appetite for them […] No appetite, because they are always full of milk."*

For peer review only

	Feeding to fill the belly	Community member	*"I have heard comments like breast milk is too thin, it digests too quickly so that they need formula that will make their belly fuller as well, that is another indication that I have seen that they look for the belly to be sort of bulging a little bit."*
		Community member	*"There have been times, many, many times we've been telling parents how their baby's stomach is probably this small, like the size of their fist, that's how big your stomach is. So, if you are trying to feed them that much rice on a plate, that is more than they should be taking in. Parents tend to think oh no she didn't eat properly, she hasn't had 5 bites and they want to give more bites in her mouth or encourage to eat more. But really that is all the food she can take."*
		Mother	*"I never know, my husband always says that's enough, don't give no more. He likes you know Asians make this thing called Kheer, it is milk with rice and sugar and it is really sweet and it is really nice. He absolutely loves it. Any time of day you feed him that he'll eat it."*
		Mother	*"I just don't want him to have too much sweet, because obviously all the kids in my family had all teeth taken out. I don't want him to go through that, he's only*

For peer review only

		got five. I don't want him to lose all his beautiful teeth. I try and keep him away from sweets. I say that but I want to keep him away from sweet stuff"
	Community member	*"[…] they are all doing it you know and if we look at infants, I have seen them with bottles drinking it. I've seen the juice, I've seen coke in and like I said, to the schools I work in they are in the buggy and they have got a bottle of brown drink and we know that it is nothing but coke, so I almost feel sorry […]"*
	Mother	*"You put him in his highchair and give him a few spoons and that's it. No, turns his face. And I have to like literally beg him, please one more, one more, he won't eat. And he's quite a healthy child. You'd think he eats quite a bit, he doesn't. He drinks 8ozs of milk and for him that's enough, he doesn't want no more."*
	Mother	*"[…] especially in the Bangladeshi community they have a Vitamin D deficiency so mum milk has everything but if the mum doesn't have enough vitamin D, the baby is not going to get enough vitamin D, so doctors are saying that formula milk has vitamin D, so mixed feeding is good. I talk to the doctor and my health visitor, they all listen to me. The formula milk has vitamin D specially made for the deficiency and so yes, I need to give him formula milk."*

For peer review only

Prolonged parent-led feeding practices	Community member	*"I think because my Mum's always hand feed us, even when we were quite old she would hand feed us, […] I'm sure that Mum's here have hand fed their […] my son is five years old and I will hand feed him because rice and curry is mostly hand fed. Also, it's the love as well, it's the love aspect. It's like you're showing them you love your child, so then you feed them more. More than the love aspect is the easiness […] instead of them making a mess you just feed them […]"*
	Mother	*"Culturally we've always eaten rice by hand, okay. That's something I would never let go of. In all honesty, rice and curry is so tasty with hands, we don't enjoy it with fork and knife."*
	Mother	"I saw most of the mothers running behind children with a spoon"
	Father	*"My boy is used to the bottle from his father, maybe at just one or two months he did breastfeeding, after that he didn't breast feed now he is sleeping my wife tried the breastfeeding at night time. Sometimes he drinks but sometimes not, but he used to have the bottle and it's easier for us as well to give him the bottle."*

For peer review only

	Health professional	*"A third of our children are on the SEN [special educational needs] Register which is speech and language. They are still probably having bottles, they are probably still on dummies and they probably aren't eating solid foods properly and they are being (hand) fed as well. That's the other thing in our community as well that children up until the age of about 7 or 8, even older, are physically fed rice, you know, hand fed. When children come into nursery… some of them don't even have a clue about how to use a knife and fork because they have been fed. When the food is fed… rice is mushed with their hand… it doesn't give them that whole experience of the chewing."*
	Health professional	*"It is almost a cultural thing in our society now that we let children have bottles for longer, one it is the oral health thing, you know if they are having sugary drinks via a bottle which is also wrong for their teeth, dental decay."*
Influences from society and culture	Mother	*"If we try to talk to her about [overfeeding her child] she gets offended. In Bengali culture, we have what we call the 'evil eye' […] so, she thinks we are giving her children the 'evil eye' because they eat well, they eat a lot. We still talk to her about it [overfeeding] though, we don't stop."*

For peer review only

	Key Informant	*"Another thing, which is quite true actually, here now for example right, it's a cultural thing. Back home right for example, if you got a pot belly for example, if you got, they think you are healthy."*
	Pregnant woman	*"The baby who has good health with no disease, is chubby. With good health they look nice."*
Physical and local environment	Community member	*"If you want to be healthy you've got to start from the beginning. Here what do you do? People are living in a box, just in the house or flat, got very limited space for a young child, 9 months or 1-year old child, it's time for them to crawl around. Is there any space to do that? No. Their bone structure, everything, is not getting stronger because they are not exercising, they are not moving around that much. What they do, say put them in a pushchair, in front of the TV and that's it."*
	Grandfather	*"[…] my grandchild is nearly 6 years old and we did try a little bit of chips to taste it."*
	Community member	*"[…] I think anyway if you go to the supermarket and you see something that says four months then you should be doing that because the packaging, if the baby food companies are telling you that you should be feeding your baby at*

For peer review only

***Theme two:* Factors influencing infant feeding and care practices in the British-Bangladeshi population**		*four months why wouldn't you, why would you wait until they were six months, because even though it is not that much of a difference but when you have had a baby they are growing so quickly you think that two months is a big deal and that you should be giving that extra food, but definitely I do not think many families that I have seen will wait until six months."*
	Key Informant	*"[…] Especially around holiday time everyone goes to everyone's houses and things like that. A lot of the dishes would be made, obviously when they are doing these kinds of things you are not looking at what you are cooking, what kind of ingredient is going in, what level of salt or sugar has gone in. That is when children do eat quite a lot more than what they are supposed to eat when you have got guests coming into your house they are bringing like sweets, crisps and things like that for your children."*
	Mother	*"I am always confused, I am always ringing up the doctors and asking them questions all the time, especially about the milk thing. They said it depends on your child, every child is different. I was a bit like he's still little, doesn't he need the nutrients in the powder to grow?"*

For peer review

	Mother	*"By starving, I actually used that term, starving may mean they think the child has not eaten [...] like for example if they have not eaten in two or three hours [...] I will give you a perfect example, I went to the wedding over the weekend and my mother in law was on my case to feed my son... I just said when he is hungry he can communicate with me, he will come up to me and say mum I want to eat. I tried three times, after three times I am not going to go round walking after him. My mother in law you know how she is she is so protective of her grandchildren[...] she took him and she fed him, so can you see the difference?"*
Information and awareness	Mother	*"I remember before I got married before I had my kids they used to do what my mum did which my mum did twenty-three years ago, twenty four years ago, at that time it was just egg custard and stuff so they did not know much about vegetables, they did now know much about a lot of stuff. Even when we were growing up probably my mum gave like at six months they started introducing what they ate but before that they gave more like egg custards"*

For peer review only

	Grandmother	*"[If] they are fussy eater, it is just because they were fed on only milk because of my daughter's in laws, they are afraid that house will be a mess. They do not know the foods, you have to try foods to know."*
	Mother	*"Yes, I am worried about him because he's not eating. I try and give him, if he eats anything like the other day I gave him a choc chip roll when I shouldn't have, like it's not good for him to have and he ate it and I was so happy. He never says no to chocolate though, that's a bad thing. Milky bar, his grandparents, always milky bars, he loves it."*
	Mother	*"I notice in our community we have a real thing about feeding them and making sure the fact they are having a full ... do you see what I mean but I just tend to find that be a bit relaxed, see if they take to it, if they eat or whatever. If they don't eat, come back to it, come back to the same meal, come back to that thing. It might be you try something else, they might like it but don't become stressed by it."*

For peer review only

Reporting checklist for qualitative study.

Based on the SRQR guidelines.

Instructions to authors

Complete this checklist by entering the page numbers from your manuscript where readers will find each of the items listed below.

Your article may not currently address all the items on the checklist. Please modify your text to include the missing information. If you are certain that an item does not apply, please write "n/a" and provide a short explanation.

Upload your completed checklist as an extra file when you submit to a journal.

In your methods section, say that you used the SRQRreporting guidelines, and cite them as:

O'Brien BC, Harris IB, Beckman TJ, Reed DA, Cook DA. Standards for reporting qualitative research: a synthesis of recommendations. Acad Med. 2014;89(9):1245-1251.

		Reporting Item	Page Number
**Title**			
	#1	Concise description of the nature and topic of the study identifying the study as qualitative or indicating the approach (e.g. ethnography, grounded theory) or data collection methods (e.g. interview, focus group) is recommended	1,3
**Abstract**			
	#2	Summary of the key elements of the study using the abstract format of the intended publication; typically includes background, purpose, methods, results and conclusions	3
**Introduction**			
Problem formulation	#3	Description and signifcance of the problem / phenomenon studied: review of relevant theory and empirical work; problem statement	5,6
Purpose or research question	#4	Purpose of the study and specific objectives or questions	6,7

For peer review only

Methods

Qualitative approach and research paradigm	#5	Qualitative approach (e.g. ethnography, grounded theory, case study, phenomenolgy, narrative research) and guiding theory if appropriate; identifying the research paradigm (e.g. postpositivist, constructivist / interpretivist) is also recommended; rationale. The rationale should briefly discuss the justification for choosing that theory, approach, method or technique rather than other options available; the assumptions and limitations implicit in those choices and how those choices influence study conclusions and transferability. As appropriate the rationale for several items might be discussed together.	7
Researcher characteristics and reflexivity	#6	Researchers' characteristics that may influence the research, including personal attributes, qualifications / experience, relationship with participants, assumptions and / or presuppositions; potential or actual interaction between researchers' characteristics and the research questions, approach, methods, results and / or transferability	7,8
Context	#7	Setting / site and salient contextual factors; rationale	8
Sampling strategy	#8	How and why research participants, documents, or events were selected; criteria for deciding when no further sampling was necessary (e.g. sampling saturation); rationale	8,9
Ethical issues pertaining to human subjects	#9	Documentation of approval by an appropriate ethics review board and participant consent, or explanation for lack thereof; other confidentiality and data security issues	10,11
Data collection methods	#10	Types of data collected; details of data collection procedures including (as appropriate) start and stop dates of data collection and analysis, iterative process, triangulation of sources / methods, and modification of procedures in response to evolving study findings; rationale	8,9
Data collection instruments and technologies	#11	Description of instruments (e.g. interview guides, questionnaires) and devices (e.g. audio recorders) used for data collection; if / how the instruments(s) changed over the course of the study	10
Units of study	#12	Number and relevant characteristics of participants, documents, or events included in the study; level of participation (could be	12

For peer review only

		reported in results)	
Data processing	#13	Methods for processing data prior to and during analysis, including transcription, data entry, data management and security, verification of data integrity, data coding, and anonymisation / deidentification of excerpts	10
Data analysis	#14	Process by which inferences, themes, etc. were identified and developed, including the researchers involved in data analysis; usually references a specific paradigm or approach; rationale	11
Techniques to enhance trustworthiness	#15	Techniques to enhance trustworthiness and credibility of data analysis (e.g. member checking, audit trail, triangulation); rationale	7,8
**Results/findings**			
Syntheses and interpretation	#16	Main findings (e.g. interpretations, inferences, and themes); might include development of a theory or model, or integration with prior research or theory	12-29
Links to empirical data	#17	Evidence (e.g. quotes, field notes, text excerpts, photographs) to substantiate analytic findings	14-29
**Discussion**			
Intergration with prior work, implications, transferability and contribution(s) to the field	#18	Short summary of main findings; explanation of how findings and conclusions connect to, support, elaborate on, or challenge conclusions of earlier scholarship; discussion of scope of application / generalizability; identification of unique contributions(s) to scholarship in a discipline or field	31-35
Limitations	#19	Trustworthiness and limitations of findings	32
**Other**			
Conflicts of interest	#20	Potential sources of influence of perceived influence on study conduct and conclusions; how these were managed	36
Funding	#21	Sources of funding and other support; role of funders in data collection, interpretation and reporting	7, 36

None The SRQR checklist is distributed with permission of Wolters Kluwer © 2014 by the Association of American Medical Colleges. This checklist can be completed online using https://www.goodreports.org/, a tool made by the EQUATOR Network in collaboration with Penelope.ai

For peer review only
